# Phenolic Profiles and Bioactivities of Ten Original Lineage Beans in Thailand

**DOI:** 10.3390/foods11233905

**Published:** 2022-12-03

**Authors:** Chaowanee Chupeerach, Piya Temviriyanukul, Sirinapa Thangsiri, Woorawee Inthachat, Yuraporn Sahasakul, Amornrat Aursalung, Pitthaya Wongchang, Parichart Sangkasa-ad, Aphinya Wongpia, Auytin Polpanit, Onanong Nuchuchua, Uthaiwan Suttisansanee

**Affiliations:** 1 Food and Nutrition Academic and Research Cluster, Institute of Nutrition, Mahidol University, Salaya, Phuttamonthon, Nakhon Pathom 73170, Thailand; 2 Biotechnology Research and Development Office, Department of Agriculture Rangsit-Nakorn Nayok, Rangsit (Klong 6), Thanyaburi, Pathum Thani 12100, Thailand; 3 Chiang Mai Field Crops Research Center, Department of Agriculture, Nong Han, San Sai District, Chiang Mai 50290, Thailand; 4 National Nanotechnology Center (NANOTEC), National Science and Technology Development Agency (NSTDA), Klong Luang, Pathum Thani 12120, Thailand

**Keywords:** enzyme inhibition, isoflavones, *Glycine max*, *Phaseolus lunatus*, *Vigna angularis*, *Vigna radiata*, *Vigna mungo*, *Vigna umbellata*

## Abstract

Legumes and pulses are important food components with various phytochemicals and health benefits. However, the health-related bioactivities of some underutilized species remain uninvestigated. To breed a new bean lineage with particular health-related properties, this study investigated phenolics (specifically, isoflavones) and the in vitro inhibitory activities of the enzyme relevant to some non-communicable diseases in underutilized cultivars of *Phaseolus lunatus* (lima beans), compared to the commonly consumed *P. vulgaris* (red kidney bean) and beans in the *Glycine* and *Vigna* genera. The results indicated that soybeans in the *Glycine* genus contained the highest isoflavone contents, especially glycitein (1825–2633 mg/100 g bean) and daidzein (1153–6471 mg/100 g bean), leading to potentially higher enzyme inhibitory activities (25–26% inhibition against α-amylase, 54–60% inhibition against α-glucosidase, 42–46% inhibition against dipeptidyl peptidase IV, 12–19% inhibition against acetylcholinesterase and 20–23% inhibition against butyrylcholinesterase) than those from other genera. Interestingly, lima beans with low isoflavone content (up to 2 mg/100 g bean) still possessed high inhibitory activities against lipase (12–21% inhibition) and β-secretase (50–58% inhibition), suggesting that bioactive compounds other than the isoflavones might be responsible for these activities. Isoflavone contents and enzyme inhibitory activities in *Vigna* beans were diverse, depending on the particular cultivars. The information gained from this study can be used for further investigation of bioactive components and in-depth health properties, as well as for future breeding of a new lineage of bean with specific health potentials.

## 1. Introduction

Beans in the Leguminosae family are one of the most widely eaten global economic plants; they are inexpensive, nutrient-dense, genome-safe [1] and can be developed into many dishes. Based on bean consumption per capita in 2019, Rwanda was found to be the country with the highest bean consumption (34.8 kg), followed by Burundi, while other investigated countries consumed less than half of that of Rwanda [2]. A survey conducted by Iowa State University (Ames, IA, USA) indicated that the most frequent bean consumption was 2–3 times per month (42.3%), followed by 1–2 times per week (29.6%) [3]. Beans are well known for their high protein, dietary fiber and minerals such as calcium and potassium [4]. Beans are also rich in nutritive and non-nutritive components, with considerable health-promoting properties. Seventy-two phytochemicals were detected in different varieties of green beans (*Phaseolus vulgaris* L.) cultivated in Spain, utilizing high-performance liquid chromatography coupled with electrospray time-of-flight mass spectrometry (HPLC-ESI-TOF-MS) [5]. These were mostly flavonoids including kaempferol, quercetin and their derivatives [5]. Four phenolic acids (caffeic acid, *p*-coumaric acid, ferulic acid and syringic acid) and two flavonoids (vitexin and isovitexin) were dominantly found in mung beans (*Vigna radiate* L.) harvested from different regions in China [6]. Furthermore, isoflavones, which are subtypes of flavonoids that resemble the sex hormone, estrogen (phytoestrogens), have also been identified in beans, such as daidzein, daidzin, glycitein, glycitin, genistein and genistin [7]. Worldwide, phytoestrogens are found at up to 4–5 mg/g dry weight, particularly in green beans, mung beans and soybeans [7], suggesting high amounts of these phytochemicals compared to other edible plants. Information on the isoflavone contents in twenty-three soybeans led to the breeding of new soybean lineage genotypes with high isoflavone contents [8]. From this information, internal (i.e., species and maturity) and external (i.e., growth environment and detection method) factors might affect the type and quantity of phytochemicals detected in beans.

Phenolics are non-nutritive compounds of great interest for clinical nutrition due to their wide range of positive health effects, such as antioxidant, anti-cancer and anti-Alzheimer’s disease (AD). The World Health Organization (WHO) (2021) stated that non-communicable diseases (NCDs) accounted for 71% of all fatalities worldwide, or 41 million deaths annually [9]. Interestingly, low- and middle-income nations, including Thailand, account for 77% of all fatalities caused by NCDs. The treatment of NCDs and palliative care were mentioned by the WHO as important aspects of the response to NCDs [9]. Hence, to reduce the risk of NCDs in low- and middle-income countries, the intake of economical and widely available foods with anti-NCD effects such as beans is a possible alternative approach. Copious evidence has supported the health benefits of beans. Oral administration of 50 mg/kg body weight of purified white kidney beans (*Phaseolus vulgaris* L.), which exhibited an α-amylase inhibitor, along with starch to adult rats resulted in significantly lower blood sugar levels compared to the control group, indicating the anti-diabetic effects of beans via the inhibition of the starch digesting enzyme, α-amylase [10]. A daidzein derivative also showed anti-obesity properties by reducing pancreatic lipase activities, leading to reduced total plasma cholesterol, low-density lipoproteins and free fatty acids in high-fat diet-treated mice [11]. The inhibition of enzymes implicated in the development of NCDs is the primary mechanism through which beans lower disease risk.

Bioactive compounds and health-related information have been widely reported in the most commonly consumed beans but some underutilized beans are missing these data, leading to low market value and reduced use in food applications. Scant information is also available on breeding new lineages within the same genera with particular properties. Previously, we reported the nutritive values and antioxidant activities of ten original lineage beans collected by the Genebank Research and Development Group, Biotechnology Research and Development Office, Ministry of Agriculture and Cooperatives, Thailand, in underutilized lima beans and commonly consumed beans including red kidney bean, red bean, azuki bean, black gram, mung bean and soybean [4]. To further explore the bioactive components and health-promoting properties of these ten beans, especially underutilized lima beans, this research aimed to investigate their phytochemical contents and enzyme inhibitory activities. The phytochemicals were determined regarding phenolics and isoflavones, while the health-related properties against some NCDs were performed through the in vitro inhibition of key enzymes including lipase (obesity), α-glucosidase, α-amylase and dipeptidyl peptidase-IV (diabetes), as well as acetylcholinesterase, butyrylcholinesterase and β-secretase (AD).

## 2. Materials and Methods

### 2.1. Sample Selection, Preparation and Extraction

Ten bean cultivars collected in the Genebank Research and Development Group, Biotechnology Research and Development Office, Department of Agriculture (DOA), Thailand, were planted in the research field of Chiang Mai Field Crops Research Center, Department of Agriculture, Chiang Mai, Thailand. Bean seeds were collected in April 2020 with physical appearances shown in Supplementary Table S1. Voucher specimens were assigned by the Bangkok Herbarium (BK), Bangkok, Thailand, including *Phaseolus lunatus* L. cultivar ‘38’ (BK083065), *P. lunatus* L. cultivar ‘47’ (BK083066), *P. lunatus* L. cultivar ‘59’ (BK083064), *P. vulgaris* L. cultivar ‘112’ (BK083063), *Vigna umbellata* (Thunb.) Ohwi and H. Ohashi cultivar ‘107’ (BK083067), *V. angularis* (Wild.) Ohwi and Ohashi cultivar ‘108’ (BK083062), *V. mungo* (L.) Hepper cultivar ‘CN4’ (BK083061), *V. radiata* (L.) Wilczek cultivar ‘CN84-1’ (BK083072), *Glycine max* (L.) Merrill cultivar ‘SJ5’ (BK083060) and *G. max* (L.) Merrill cultivar ‘CM60’ (BK083057). All beans were ground into fine powder using a grinder (600W series from Philips Electronics Co., Ltd., Jakarta, Indonesia). The powdery samples were kept at −20 °C until analysis.

The extraction of bean samples followed previous research [4]. Briefly, the powdery bean sample (1 g) was mixed with 70% (*v*/*v*) ethanol (20 mL) before shaking in a 30 °C water bath shaker (WNE45 series from Memmert GmBh, Eagle, WI, USA) for 2 h. The mixture was then centrifuged at 3800× *g* using a refrigerated centrifuge (Hettich^®^ ROTINA 38R from Andreas Hettich GmbH, Tuttlingen, Germany) for 15 min. The supernatant was collected and filtered through 0.45 µM polyethersulfone membrane syringe filter for further analysis.

### 2.2. Determination of Phenolics Utilizing Liquid Chromatography-Electrospray Ionization Tandem Mass Spectrometry

#### 2.2.1. Preparation of the Lineage Beans for Phenolic Analysis

The supernatant collected in Section 2.1 was freeze-dried using a −50 °C freeze-dryer (Heto Powerdry PL9000 from Heto Lab Equipment, Allerod, Denmark) at 0.086 mbar for 72 h. The powdery bean extract (0.5 g) was dissolved in an acidic methanol solution (prepared by mixing 40 mL formic acid with 10 mL 62.5% (*v*/*v*) methanol containing 0.5 g *tert*-butylhydroquinone) and shaken in an 80 °C water bath shaker (TW20 series from Julabo GmbH, Seelbach, Germany) for 2 h. To stop the reaction, the mixture was incubated on ice for 5 min before adding 1% (*v*/*v*) ascorbic acid (100 µL). Sonication utilizing an ultrasonic bath (Ultrasonics™ M series from Branson Ultrasonics Corp., Danbury, CT, USA) was performed for 5 min. Then, the volume of the mixture was adjusted to 50 mL using 62.5% (*v*/*v*) methanol containing 0.5 g *tert*-butylhydroquinone and filtered through a 0.22 µM polytetrafluoroethylene membrane syringe filter. The filtrate was loaded onto a liquid chromatography-electrospray ionization tandem mass spectrometry (LC-ESI-MS/MS) system as described below.

#### 2.2.2. Screening of Phenolics

The LC-ESI-MS/MS system was used to identify phenolics in the lineage beans. The system was equipped with a Dionex Ultimate 3000 series ultrahigh-performance liquid chromatographer (UHPLC), a TSQ Quantis Triple Quadrupole mass spectrometer (MS) and a diode array detector with a Chromeleon 7 chromatography data system (version 7.2.9.11323) from Thermo Fisher Scientific (Bremen, Germany).

The peak retentions and mass chromatograms of the bean samples were compared with commercial standards including twelve phenolic acids and eighteen flavonoids (including six isoflavones). These standards were apigenin (>98.0% HPLC), (−)-epigallocatechin gallate (>98.0% HPLC), 3,4-dihydroxybenzoic acid (≥97% T), 4-hydroxybenzoic acid (>99.0% GC, T), hesperidin (>90.0% HPLC, T), chlorogenic acid (>98.0% HPLC, T), caffeic acid (>98.0% HPLC, T), *p*-coumaric acid (>98.0% GC, T), luteolin (>98.0% HPLC), kaempferol (>97.0% HPLC), genistein (>98.0% HPLC), myricetin (>97.0% HPLC), syringic acid (>97.0% T), ferulic acid (>98.0% GC, T), cinnamic acid (>98.0% HPLC), naringenin (>93.0% HPLC, T), quercetin (>98.0% HPLC, E) and sinapic acid (>99.0% GC, T) from Tokyo Chemical Industry (Tokyo, Japan); rutin (≥94% HPLC), gallic acid (97.5–102.5% T), vanillic acid (≥97% HPLC), rosmarinic acid (≥98% HPLC) and cinnamic acid (≥99% HPLC) from Sigma-Aldrich (St. Louis, MO, USA); galangin (≥98.0% HPLC) from Wuhan ChemFaces Biochemical Co., Ltd. (Hubei, China); isorhamnetin (≥99.0% HPLC) from Extrasynthese (Genay, France); daidzein (≥98.0% HPLC), daidzin (≥98.0% HPLC), glycitin (≥98.0% HPLC), glycitein (≥98.0% HPLC) and genistin (≥98.0% HPLC) from Tokyo Chemical Industry (Tokyo, Japan).

The reaction monitoring transitions were optimized for commercial standards. Each standard (10 µL of 20 µg/mL) was loaded onto the LC-ESI-MS/MS system using a flow rate of 0.5 µL/min with 10 min run time for phenolics and 20 min run time for isoflavones. Positive fragment ions were generated utilizing a heated electrospray ion source (HESI) and determined using a TSQ Quantis Triple Quadrupole MS with parameters as follows: 50–1000 *m*/*z* mass range, 3500 V positive ion, 30 Arb sheath gas (N_2_), 15 Arb auxiliary gas (N_2_), 325 °C ion transfer tube temperature and 350 °C vaporizer temperature. Full scanning (FS) and selective reaction monitoring (SRM) modes were selected for qualitative analysis. The MS/MS ion fragments (parent ions and SRM transitions) were achieved using specific collision energies and radio frequencies (RF-lens), as shown in Supplementary Table S2.

To create the calibration curves, a mixture of all commercial standards was prepared in methanol and diluted to different levels to obtain the linear range. The standard mixture (10 µL) was chromatographically separated using a 2.1 mm × 100 mm, 2.6 μm Accucore RP-MS column (Thermo Fisher Scientific, Bremen, Germany) and a gradient mobile phase consisting of acetonitrile (solvent A) and 0.1% (*v*/*v*) formic acid in Milli-Q water (18.2 MΩ·cm resistivity at 25 °C, solvent B) at a flow rate of 0.5 mL/min as shown in Table 1 and Table 2. All experiments were performed in triplicate on the same day (inter-day). A linear equation (*y* = *ax* ± *c*) and correlation coefficient of linear regression (*R*^2^) of each standard were obtained using the plot between concentrations (*x*) and ion peak areas (*y*).

The method for validation parameters and quantification of phenolic acids and flavonoids followed a previously report [12,13], while parameters of isoflavones/isoflavone glycosides are reported in this study (Supplementary Table S3). The chromatograms of the standards are presented in Supplementary Figures S1 and S2.

### 2.3. Determination of Enzyme Inhibition

In vitro bioactivities of ten bean extracts were performed as inhibition of the key enzymes relevant to obesity (lipase), type II diabetes (α-glucosidase, α-amylase and dipeptidyl peptidase-IV (DPP-IV)) and AD (acetylcholinesterase (AChE), butyrylcholinesterase (BChE) and β-secretase (BACE-1)) using the well-established protocols previously reported [14,15,16,17] with some modifications as indicated in Table 3. The visualization of enzyme kinetics was performed utilizing a Synergy^TM^ HT 96-well UV-visible microplate reader (BioTek Instruments, Inc., Winooski, VT, USA) and Gen 5 data analysis software. Commercially available enzyme inhibitors, including orlistat (lipase), acarbose (α-amylase) and saxagliptin (DPP-IV), were performed as positive controls. All chemicals and reagents were received from Sigma-Aldrich (St. Louis, MO, USA).

The inhibitory activity was calculated as a decline in enzyme kinetics using the following equation.

(1) $$\%\;\mathrm{inhibition}=\left(1-\;\frac{B-b}{A-a}\right)\;\times 100,$$

where *A* is the initial velocity (*V*_0_) of the reaction with enzyme but without the bean extract (control), *a* is the *V*_0_ of the reaction without enzyme and the bean extract (control blank), *B* is the *V*_0_ of the reaction with enzyme and the bean extract (sample), and *b* is the *V*_0_ of the reaction with the bean extract but without the enzyme (sample blank).

The inhibitory activity of BACE-1 was determined using a fluorescence detection as an end-point assay; thus, the percentage of inhibition was determined utilizing Equation (1) but changing *V*_0_ to fluorescence absorbance at specific wavelength.

### 2.4. Statistical Analysis

All experiments were carried out in triplicate on three independent sets of samples (*n* = 3). The results were represented as mean ± standard deviation (SD). A one-way analysis of variance (ANOVA) and a Duncan’s multiple comparison test were performed with significant differences at *p* < 0.05. The statistical analysis was performed using the Statistical Package for the Social Sciences (version 18 for Windows, SPSS Inc., Chicago, IL, USA). Principal component analysis (PCA) and hierarchical cluster analysis (HCA) of phenolics and in vitro health-related properties were analyzed using XLSTAT^®^ (Addinsoft Inc., New York, NY, USA).

## 3. Results

### 3.1. Types and Quantities of Phenolics

Among the phenolics, beans were previously reported to contain a high content of flavonoids, especially isoflavones [18,19]. Isoflavone profiles were determined by LC-ESI-MS/MS analysis using six isoflavone standards in both aglycones and glycosylated forms, including daidzein, daidzin, glycitein, glycitin, genistein and genistin and were expressed as mg/100 g extract (Table 4) and mg/100 g bean (Supplementary Table S4). The results indicated that all bean cultivars possessed different degrees and types of isoflavones (LC-ESI-MS/MS chromatograms are shown in Supplementary Figure S3). Soybeans in the *Glycine* genus, ‘SJ5’ and ‘CM60’, contained all types of the investigated isoflavones with the highest content compared to beans in the other two genera. Among aglycone isoflavones, both soybeans exhibited higher content of glycitein (143–2111-fold higher) and daidzein (352–1334-fold higher) than genistein. The content of aglycone isoflavones were also higher than their glycosylated forms (25–26-fold higher in daidzein and 13–15-fold higher in glycitein than diadzin and glycitin, respectively). However, genistein presented a lower amount than its glycoside isoflavone, genistin (3–10-fold lower).

Beans in the *Vigna* genus exhibited lower content of isoflavones than beans in the *Glycine* genus. Among the beans in the *Vigna* genus, the red rice bean ‘107’ and azuki bean ‘108’ exhibited all of the investigated isoflavones with the exception of glycitein, which was absent in both cultivars. These two cultivars also possessed higher content of daidzein than genistein (344–378-fold higher), while the content of daidzin was higher than glycitin (3–9-fold higher) and genistin (7–9-fold higher). The mung bean ‘CN84-1’ only exhibited daidzein and glycitein and their glycosides, while genistein and genistin were undetected in this cultivar. The glycitein content was higher than daidzein (1.5-fold higher). A similar result was observed with their glycosylated forms (3.1-fold higher in glycitin). Furthermore, the content of aglycones was higher than their corresponded glycosylated forms (26-fold higher in daidzein and 12.3-fold higher in glycitein). The last cultivar in this genus, black gram ‘CN4’, only contained daidzein and its glycoside, with a daidzein content 36.3-fold higher than diadzin.

Beans in the *Phaseolus* genus possessed the lowest content of isoflavones. The lima bean ‘38’ exhibited only genistein and diadzin, while daidzein was lower than the limit of detection (LOD) (4.15 µg/mL). The lima bean ‘59’ exhibited only glycitein, while the only isoflavone detected in lima bean ‘47’ was diadzin, with a content lower than the LOD (0.04 µg/mL). The red kidney bean ‘112’ exhibited similar isoflavones as the lima bean ‘38’, with detected the glycitein also lower than the LOD (0.14 µg/mL).

In addition to isoflavones, LC-ESI-MS/MS analysis using twenty-four commercial phenolic standards (twelve flavonoids and twelve phenolic acids) indicated that all of the bean types did not possess phenolic acid, while some flavonoids (other than isoflavones) were detected in low amounts (Table 5 and Supplementary Figure S4). Soybeans in the *Glycine* genus, ‘SJ5’ and ‘CM60’, exhibited high isoflavone content; however, both cultivars possessed low amounts of naringenin, while the soybean ‘CM60’ also exhibited kaempferol at levels lower than the LOD (0.122 µg/mL) [13].

Beans in the *Vigna* genus possessed various types of flavonoids but at a low content. Interestingly, the red rice bean ‘107’ exhibited a moderate amount of kaempferol and trace amounts of quercetin at levels lower than the LOD (0.05 µg/mL) [13]. The azuki bean ‘108’ and black gram ‘CN4’ exhibited detectable amounts of isorhamnetin and levels lower than the LOD content of kaempferol. The mung bean ‘CN84-1’ contained low luteolin content and lower than the LOD content of apigenin (0.127 µg/mL) [13], naringenin (0.003 µg/mL) [13] and kaempferol.

Likewise, beans in the *Phaseolus* genus also exhibited various types of flavonoids but in low amounts. The lima bean ‘38’ exhibited low isorhamnetin content and lower than the LOD contents of quercetin, naringenin and kaempferol. The lima bean ‘47’ only exhibited trace amounts of isorhamnetin, while the lima bean ‘59’ contained lower than the LOD contents of naringenin and kaempferol. The red kidney bean ‘112’ also exhibited low quercetin content and lower than the LOD content of kaempferol and isorhamnetin (0.016 µg/mL) [13].

### 3.2. In Vitro Health-Related Activities

The enzymes investigated in this study are the key targets for medicinal-based drug design to prevent and treat NCDs. All bean extracts were investigated regarding their inhibitions against these key enzymes compared to well-known enzyme inhibitors.

One medicinal targeted pathway to control obesity is through the inhibition of the lipid-degrading enzyme, lipase. The inhibition of this enzyme leads to slow digestion of lipids, resulting in a lower rate of fat accumulation within the body [20]. Using an extract concentration of 10 mg/mL, all 10 bean cultivars displayed lipase inhibitory activities ranging from 6.89 to 21.91% inhibition, with the exception of the black gram ‘CN4’ and mung bean ‘CN84-1’, in which no inhibitory activity was observed (Table 6). Among the remaining eight bean cultivars with lipase inhibitory activities, the azuki bean ‘108’ and red kidney bean ‘112’ presented the highest inhibition, while the red rice bean ‘107’ presented the lowest. Comparing among genera, the beans in the *Phaseolus* genus exhibited higher inhibitory activities than soybeans in the *Glycine* genus and the red rice bean ‘107’ in the *Vigna* genus. However, no clear trend in lipase inhibition was observed in the *Vigna* genus that contained both the highest inhibitory provider, azuki bean ‘108’ and the lowest red rice bean ‘107’.

For control of type II diabetes, inhibition of carbohydrate-hydrolyzing enzymes, α-amylase and α-glucosidase, leads to low glucose production and absorption into the body, [23,24]. Another pathway is through the inhibition of DPP-IV, resulting in increased insulin secretion to reduce postprandial and fasting hyperglycemia [25]. Using an extract concentration of 12.5 mg/mL, all 10 bean cultivars displayed α-amylase inhibitory activities ranging from 12.53 to 31.06% inhibition with the lima bean ‘38’ and red rice bean ‘107’ exhibiting the highest activities, while the red kidney bean ‘112’, azuki bean ‘108’ and black gram ‘CN4’ exhibiting the lowest activities (Table 6). No clear trend in α-amylase inhibitory activities was observed among genera. Lima beans in the *Phaseolus* genus, ‘38’, ‘47’ and ‘59’ exhibited high inhibitory activities (25.04–31.06%), while the red kidney bean ‘112’ in the same genus exhibited lower inhibition (14.15%). Both soybeans in the *Glycine* genus exhibited similar lipase inhibitory activities (24.77–26.33%), compatible with the lima beans ‘47’ and ‘59’. Likewise, beans in the *Vigna* genus exhibited distinctive degrees of inhibition. The red rice bean ‘107’ exhibited the highest inhibition, similar to the lima bean ‘38’, while the azuki bean ‘108’ and black gram ‘CN4’ possessed the lowest inhibitory activities, similar to the red kidney bean ‘112’ in the *Phaseolus* genus. For α-glucosidase inhibitory activities, all 10 bean cultivars exhibited 6.43–59.83% inhibition using an extract concentration of 12.5 mg/mL (Table 6). Among the ten cultivars, the soybean ‘SJ5’ exhibited the highest α-glucosidase activities, while the red kidney bean ‘112’ exhibited the lowest. Both soybean cultivars in the *Glycine* genus exhibited higher α-glucosidase inhibitory activities than beans in the *Vigna* (1.1–2.2-fold higher) and *Phaseolus* (1.2–9.3-fold higher) genera. Other than the carbohydrate degrading enzymes, all 10 bean cultivars also exhibited DPP-IV inhibitory activities ranging from 11.95 to 51.62% inhibition using extract concentration of 12.5 mg/mL (Table 6). Among the ten cultivars, the black gram ‘CN4’ exhibited the highest DPP-IV activities, while the red kidney bean ‘112’ and azuki bean ‘108’ exhibited the lowest. No clear trend was observed in the DPP-IV inhibitory activities of the different genera. Soybeans in the *Glycine* genus generally provided high inhibitions (42.44–45.62%), while beans in the *Vigna* and *Phaseolus* genera exhibited both high and low inhibitory activities. In the *Vigna* genus, the black gram ‘CN4’ provided high inhibitory activities (51.62%), while lower inhibitions were observed in the azuki bean ‘108’ (4.3-fold lower). Similarly, among the *Phaseolus* beans, the lima bean ‘59’ exhibited higher inhibitory activity (47.33%) than lima beans ‘38’ and ‘47’ (1.2–1.3-fold higher) and the red kidney bean ‘112’ (3.6-fold higher).

The main hypotheses for medicinal targeted AD treatment are through increased cholinergic neurotransmitters and lower β-amyloid formation. In the cholinergic hypothesis, the inhibition of AChE and BChE increases the neurotransmitter concentrations [26], while the inhibition of BACE-1 leads to retardment of β-amyloid plaque and improvement of brain cognitive function [27]. At a concentration of 10 mg/mL, only the soybeans ‘SJ5’ and ‘CM60’ in the *Glycine* genus exhibited AChE inhibitory activities with 11.76 and 18.76% inhibitions, respectively (Table 6). No AChE inhibitory activity in other beans in the *Phaseolus* and *Vigna* genera was observed. However, using the same extraction concentration as the AChE inhibitory assay, all 10 bean cultivars exhibited BChE inhibitory activities ranging from 8.25 to 23.12%, with the soybean ‘SJ5’ exhibiting the highest and the mung bean ‘CN84-1’ the lowest (Table 6). Soybeans in the *Glycine* genus exhibited higher inhibitory activities than beans in the *Phaseolus* (1.3–1.9-fold higher) and *Vigna* (1.7–2.8-fold higher) genera. Likewise, using an extraction concentration of 2 mg/mL, all 10 bean cultivars exhibited BACE-1 inhibitory activities ranging from 16.38 to 58.23% (Table 6). The lima bean ‘38’ and black gram ‘CN4’ exhibited the highest BACE-1 inhibitions, while the azuki bean ‘108’ exhibited the lowest. All beans in the *Phaseolus* genus showed high inhibitory activities (>50%), while the beans in the *Vigna* genus exhibited different degrees of inhibition. In this genus, the black gram ‘CN4’ and mung bean ‘CN84-1’ exhibited high inhibitory activities (>50%), while less than half of these inhibitory activities were observed in the red rice bean ‘107’ and azuki bean ‘108’. Soybeans in the *Glycine* genus exhibited low to moderate inhibitory activities (22.95–29.83%).

### 3.3. Correlation Analysis by Principal Component Analysis (PCA) and Hierarchical Cluster Analysis (HCA)

**Figure 1:**
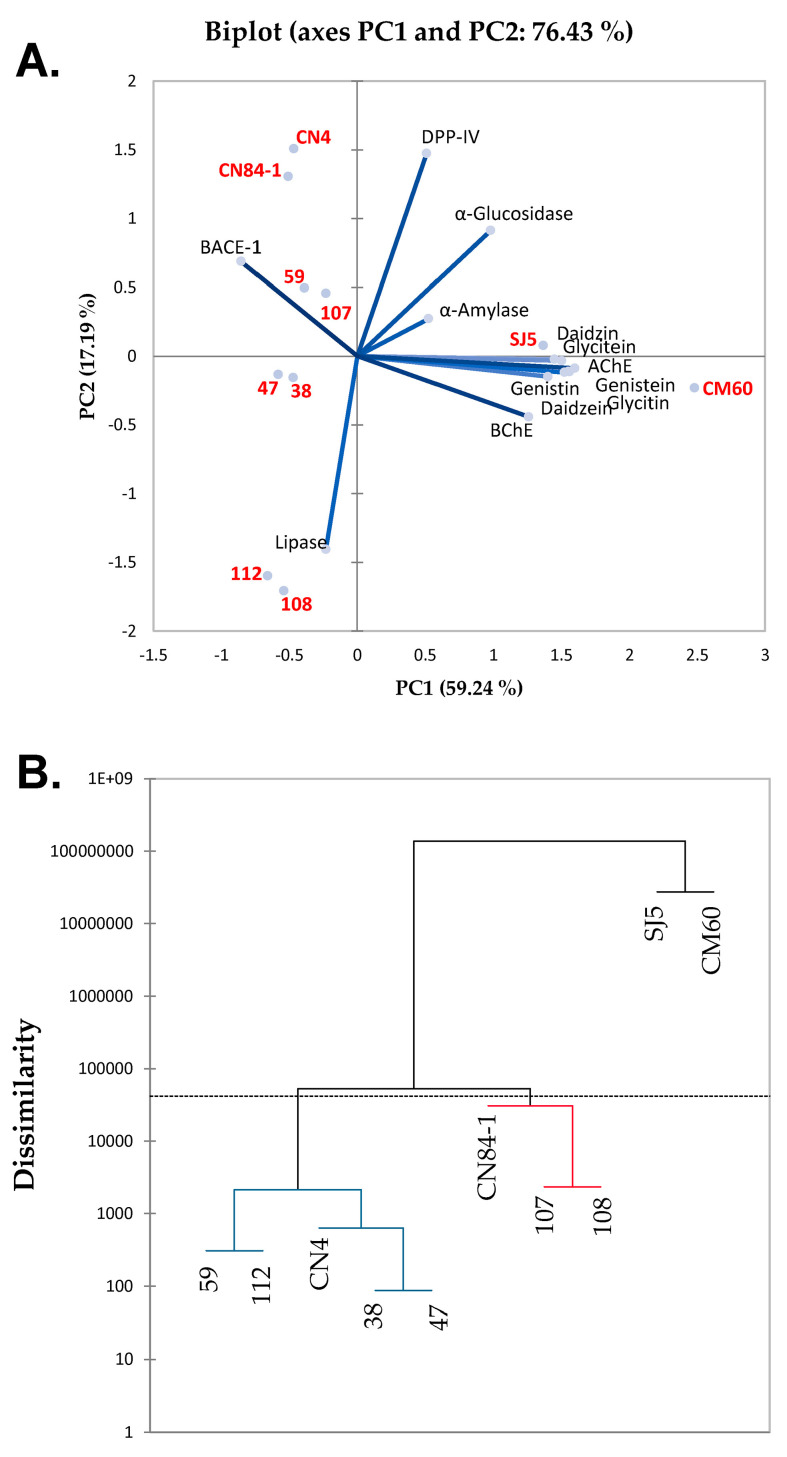
(**A**) Biplot of the principal component analysis (PCA) derived from the observations (*Phaseolus lunatus* L. cultivar ‘38’, ‘47’ and ‘59’, *Phaseolus vulgaris* L. cultivar ‘112’, *Vigna umbellata* (Thunb.) Ohwi and H. Ohashi cultivar ‘107’, *Vigna angularis* (Wild.) Ohwi and Ohashi cultivar ‘108’, *Vigna mungo* (L.) Hepper cultivar ‘CN4’, *Vigna radiata* (L.) Wilczek cultivar ‘CN84-1’ and *Glycine max* (L.) Merrill cultivar ‘SJ5’ and ‘CM60’) and variables including isoflavones and enzyme inhibitory activities against lipase, α-glucosidase, α-amylase, dipeptidyl peptidase-IV (DPP-IV), butyrylcholinesterase (BChE), acetylcholinesterase (AChE) and β-secretase (BACE-1); (**B**) dendrogram derived from the hierarchical cluster analysis (HCA) from the same data as PCA.

Principal component analysis (PCA) and hierarchical cluster analysis (HCA) were conducted to investigate the correlations between the ten bean cultivars and their properties. The mean data of the identified isoflavones and inhibitory activities against lipase, α-glucosidase, α-amylase, DPP-IV, BChE, AChE and BACE-1 were used in the PCA and HCA analyses. A biplot between the observations (ten bean cultivars) and variables (isoflavones and enzyme inhibitory activities) was generated, as shown in Figure 1A. The axes of the PCA were divided into PC1 and PC2, while the combination of PC1 and PC2 was calculated as 76.43%, implying good representation of the data. The findings revealed that soybeans in the *Glycine* genus, ‘SJ5’ and ‘CM60’, were projected together with all tested isoflavones, enzyme inhibitory activities against α-glucosidase, α-amylase, DPP-IV, AChE and BChE as a cluster. This result suggested that the soybeans, ‘SJ5’ and ‘CM60’, were rich in isoflavones with high α-glucosidase, α-amylase, DPP-IV, AChE and BChE inhibitory activities compared to the other bean cultivars, and also implied that the high amounts of isoflavones contributed to high enzyme inhibitory activities because other cultivars located far away from the tested isoflavones possessed low amounts of isoflavones. Interestingly, the red kidney bean ‘112’ and azuki bean ‘108’ were projected close to the lipase inhibitory activities, while the lima beans, ‘38’, ‘47’ and ‘59’, mung bean ‘CN84-1’ and black gram ‘CN4’ were projected close to BACE-1 inhibitory activities, suggesting particularly high enzyme inhibitory activities in these bean cultivars. However, all of these bean cultivars possessed minor amounts of isoflavones, implying that other phytochemicals in the beans may play a role in suppressing the activities of these two enzymes. 

A HCA was further used to determine clusters in all of the tested bean cultivars and beans with similar properties were grouped together. The dendrogram (Figure 1B) shows that the soybeans, ‘SJ5’ and ‘CM60’, were grouped together, implying high similarity in their isoflavones and enzyme inhibitory activities. The dissimilarity value of the soybeans, ‘SJ5’ and ‘CM60’, was also remarkably higher than the remaining cultivars, implying that these two soybeans were unique from the others. Thus, PCA and HCA data demonstrated that the soybeans, ‘SJ5’ and ‘CM60’, were high in isoflavones and enzyme inhibitory activities, specifically α-glucosidase, α-amylase, DPP-IV, AChE and BChE.

## 4. Discussion

Original lineage beans in Thailand were reported as a rich source of nutrients, phenolics and antioxidant activities that varied according to genera [4]. These beans also exhibited various bioactivities, especially health-related properties against NCDs. In this study, ten bean cultivars including (1) *Phaseolus* beans (*P. lunatus* L. or lima bean cultivars ‘38’, ‘47’ and ‘59’, and *P. vulgaris* L. or red kidney bean cultivar ‘112’), (2) *Vigna* beans (*V. umbellata* (Thunb.) Ohwi and H. Ohashi or red bean cultivar ‘107’, *V. angularis* (Wild.) Ohwi and Ohashi or azuki bean cultivar ‘108’, *V. mungo* (L.) Hepper or black gram cultivar ‘CN4’, and *V. radiata* (L.) Wilczek or mung bean cultivar ‘CN84-1’) and (3) *Glycine* beans (*G. max* (L.) Merrill or soybean cultivars ‘SJ5’ and ‘CM60’) were investigated regarding their bioactive compounds (especially isoflavones) and in vitro inhibitory activities against the key enzymes relevant to some NCDs. The results demonstrated that soybeans in the *Glycine* genus, ‘SJ5’ and ‘CM60’, contained the highest contents of isoflavones, while traces of other phenolics were detected in all genera. With different amounts of isoflavones, the soybeans also exhibited high inhibitory activities against α-amylase, α-glucosidase, DPP-IV, AChE and BChE. By contrast, the *Phaseolus* beans exhibited high inhibitory activities against lipase and BACE-1, while no clear trends in the isoflavone contents and enzyme inhibitory activities were observed in the *Vigna* beans.

Soybeans are good sources of isoflavones. Our results indicated that two soybeans, ‘SJ5’ and ‘CM60’, contained exceptionally high content of isoflavones, especially daidzein and glycitein, and concurred with previous literature, suggesting that soybeans are excellent isoflavone providers [18,19]. In our study, higher isoflavone content was detected (3193 and 9630 mg/100 g beans in soybean ‘SJ5’ and ‘CM60’, respectively, as shown in Supplementary Table S4) compared to previous reports. The isoflavone content varied depending on both internal (cultivar and maturity stage) and external (climate, temperature, soil quality and detection method) factors. Six isoflavones were detected in three different green bean varieties collected in Spain using HPLC-ESI-TOF-MS [5]. A total of 40 soybean cultivars from various ecotype regions and maturity stages in China had total isoflavone contents ranging from 55.1 to 758.4 mg/100 g as determined by HPLC analysis [28]. While using the same detection method, total isoflavone content ranging from 0.69 to 5.42 mg/g dry matter were detected in 23 soybeans at 4 different maturity stages from Serbia [8], while vitexin and isovitexin were dominantly detected in 24 mung bean genotypes collected in different regions of China [6]. Globally, the average isoflavone content was 154.53 mg/100 g soybean [29]. Our soybeans, however, exhibited distinctly high isoflavone content in a range from 10.9 to 16.6 g/100 g soybean (Supplementary Table S4). Furthermore, the content of the isoflavones and their glycoside derivatives were recorded as 40–60% genistin/genestein, 30–50% diadzin/diadzein and 12–13% glycetin/glycitein of total isoflavones [19,30]. The opposite result was observed in this study, with the content of genistin/genestein, diadzin/diadzein and glycetin/glycitein in the soybean ‘SJ5’ of 0.3%, 38.0% and 61.7% of total isoflavones, respectively, while those of the soybean ‘CM60’ were 0.8%, 69.7% and 29.4% of total isoflavones, respectively. Thus, the differences in the types and quantity of isoflavones depended on both internal and external factors as stated above.

Due to the distinguished amounts of isoflavones, soybeans exhibited high inhibitory activities against α-glucosidase, α-amylase, DPP-IV, AChE and BChE. The inhibition of carbohydrate-degrading enzymes, α-glucosidase and α-amylase, slows down the rate of glucose absorption into blood vessels, thus maintaining circulating glucose levels [24]. Phenolics act as effective inhibitors against both α-glucosidase and α-amylase; however, a stronger inhibitory effectiveness was detected on α-glucosidase than α-amylase [31]. Among phenolics, flavonoids act as stronger inhibitors than phenolic acids, tannin and coumarin [31,32]. As one sub-class of flavonoids, isoflavones, especially diadzein and glycitein the two main isoflavones detected in our soybeans, were possibly responsible for the α-amylase and α-glucosidase inhibitory activities. The IC_50_ values of daidzein against α-amylase and α-glucosidase were 301 and 48 µM, respectively, and stronger than acarbose, a competitive inhibitor of α-amylase and α-glucosidase [33]. However, no information on α-amylase and α-glucosidase inhibition is available for glycitein. Another pathway to control type II diabetes is through the inhibition of DPP-IV, the enzyme that degrades incretin hormones including GLP-1 (glucagon-like peptide-1) and GIP (gastric inhibitory peptide). These two hormones maintain glucose homeostasis by elevating insulin secretion, lowering glucagon concentrations and delaying gastric emptying. Thus, DPP-IV inhibition increased incretin hormone levels, leading to increased insulin secretion and reducing postprandial and fasting hyperglycemia. No previous report on the effect of daidzein and glycitein on anti-DPP-IV activity is available; however, streptozotocin-induced diabetic mice and normal mice treated with daidzein (10 mg/kg body weight) showed significantly reduced postprandial blood glucose levels [33]. Interestingly, only women with high plasma isoflavone contents were found to be associated with lower risk of type II diabetes [34], while in men, serum isoflavone levels were unrelated to type II diabetes [34]. Similar results were observed in overweight women with a body mass index (BMI) ≥ 25 kg/m^2^ who had a high soy product intake and lower risk of type II diabetes development [35]. Adults with a high intake of soy food and isoflavone (genistein, daidzein and glycitein) were also found to be associated with a lower risk of type II diabetes [36]. Thus, the consumption of soybeans with high isoflavone content can reduce the risk of type II diabetes.

One hypothesis of AD occurrence is cholinergic deficiency, involving over-activity of two cholinesterase enzymes, AChE and BChE, which are neurotransmitter, acetylcholine, degrading enzymes. Thus, over-active cholinesterases can lead to insufficient neurotransmission, deterioration of cholinergic neurons and finally cognitive decline [26]. No information is available on anti-AChE and -BChE activities of both daidzein and glycitein. However, by using passive avoidance and Morris water maze tests, the administration of daidzin or daidzein (5 mg/kg) improved cognitive dysfunction induced by cholinergic blockade by acting on estrogen receptors in scopolamine-induced learning and memory impaired mice [37]. Daidzein metabolite or 6,7,4-trihydroxyisoflavone (6,7,4-THIF) also reduced hippocampus AChE activity and improved learning memory deficits in the cholinergic nervous system of scopolamine-induced learning and memory impaired mice [38]. Soy isoflavones also ameliorated memory deficits from the loss of cholinergic input (cholinergic degeneration), and reduced age-related neuronal loss and cognition decline in elderly male rats [39].

Even though the lima bean ‘38’, ‘47’ and ‘59’ contained trace amount of isoflavones, these beans exhibited high inhibitory activities against lipase and BACE-1. Compared to the other 12 bean cultivars, low TPC in the lima bean was also previously reported as 1.17 mg GAE/g bean, leading to trace lipase inhibitory activity [40]. While only one available study in the literature reported lipase inhibitory efficacy of the lima bean [40], this result was opposite to ours, suggesting that other bioactive compounds in the lima bean might contribute to the high lipase inhibition in our study. One possibility is that active proteins/peptides, as previously reported for Pinto bean peptides with a particular amino acid sequence, could effectively inhibit lipase activity [41]. Furthermore, feeding protein isolated from cowpea seed was found to decreased total plasma cholesterol [42].

A similar result was observed in BACE-1 inhibitory activity of our lima beans. Strong BACE-1 inhibitions were detected, while these beans exhibited low isoflavone content. Other bioactive components such as active proteins/peptides might be responsible for these inhibitory activities. BACE-1 inhibitors can be divided into peptidic and non-peptidic groups [43,44]. Several peptidic inhibitors are derived from natural sources [45,46]; however, none were previously reported regarding bean peptide. Saponin was found in moderated amounts in the lima bean [40], while ginseng saponin reduced BACE-1 activity [47,48] but was not reported for bean saponin. These hypotheses required further investigation to confirm the types of bioactive compounds (other than phenolics) in lima beans responsible for BACE-1 inhibitory activities.

This study demonstrated that soybeans in the *Glycine* genus that were rich in isoflavones exhibited high α-glucosidase, α-amylase, DPP-IV, AChE and BChE inhibitory activities compared to other bean cultivars. These results implied that isoflavones might potentially act as enzyme inhibitors. However, lima beans in the *Phaseolus* genus exhibited high lipase and BACE-1 inhibitory activities even though they contained trace amounts of isoflavones, suggesting that other bioactive compounds such as active peptide/protein might be responsible for these activities.

## 5. Conclusions

Beans have been widely reported for their bioactive compounds and health-promoting properties but information is lacking, especially on the underutilized lima beans. This study investigated phenolics, especially isoflavones, and inhibitory activities against the key enzymes relevant to the control of obesity (lipase), diabetes (α-amylase, α-glucosidase and DPP-IV) and AD (AChE, BChE and BACE-1). The soybean ‘SJ5’ and ‘CM60’ exhibited relatively high enzyme inhibitory activities against α-glucosidase, α-amylase, DPP-IV, AChE and BChE, possibly due to their exceptionally high isoflavone contents, especially daidzein and glycitein. Interestingly, lima beans exhibited high lipase and BACE-1 inhibitory activities even though they contained low isoflavone contents, suggesting that other bioactive compounds might be responsible for these activities. *Vigna* beans, on the other hand, exhibited diverse enzyme inhibitory activities and isoflavone contents, depending on the particular cultivars.

Our results provide a valuable reference for further investigation of bioactive components (other than phenolics) and in-depth health properties of underutilized lima beans. The information from this study can also be used to benefit other bean cultivars and promote the fulfillment of missing health information by further comprehensive investigation of cell culture and in vivo or clinical studies. The investigation of the bioaccessibility of these beans and the bioactivities of the digested beans would be an interesting direction for future work. This information can support future applications in functional food development and breeding strategy within the same genus to produce new progeny with both high bioactive agents and advantageous health properties.

## Figures and Tables

**Table 1:** A gradient mobile phase of liquid chromatography-electrospray ionization tandem mass spectrometry for phenolic analysis.

Time (min)	%Solvent A	%Solvent B	Flow Rate (mL/min)
0.0	10	90	0.5 mL/min
8.0	80	20	
8.1	10	90	
10.0	10	90	

Solvent A: acetonitrile; solvent B: 0.1% (*v*/*v*) formic acid in Milli-Q water.

**Table 2:** A gradient mobile phase of liquid chromatography-electrospray ionization tandem mass spectrometry for isoflavone analysis.

Time (min)	%Solvent A	%Solvent B	Flow Rate (mL/min)
0.0	5	95	0.5 mL/min
2.0	5	95	
5.0	50	50	
7.0	50	50	
10.0	90	10	
13.0	90	10	
15.0	5	95	
20.0	5	95	

Solvent A: acetonitrile; solvent B: 0.1% (*v*/*v*) formic acid in Milli-Q water.

**Table 3:** The assay components for enzyme inhibitory assays.

Assay	Assay Components				
	Enzyme	Substrate	Indicator	Extract	Detection Wavelength
Lipase	100 µL of 20 µg/mL lipase ^1^	50 μL of 0.2 mM DNPDB	10 µL of 16 mM DTNB	40 µL	412 nm
AChE	100 μL of 0.25 µg/mL AChE ^2^	50 μL of 0.32 mM ACh			
BChE	100 μL of 1.5 µg/mL BChE ^3^	50 μL of 0.4 mM BCh			
α-Amylase	100 µL of 50 mg/mL α-amylase ^4^	50 µL of 30 mM pNPM		50 µL	405 nm
α-Glucosidase	100 µL of 0.1 U/mL α-glucosidase ^5^	50 µL of 2 mM pNPG		50 µL	
DPP-IV	50 µL of 0.02 U/mL DPP-IV ^6^	50 µL of 12 mM Gly-Pro-pNA + 50 µL 100 mM Tris-HCl (pH 8)		50 µL	
BACE-1	BACE-1 FRET assay kit (Sigma-Aldrich, St. Louis, MO, USA) following manufacturer’s recommendations				λ_ex_ = 320 nm λ_em_ = 405 nm

AChE: acetycholinesterase; BChE: butyrylcholinesterase; DPP-IV: dipeptidyl peptidase-IV; BACE-1: β-secretase; DNPDB: 5-5′-dithiobis(2-nitrobenzoic-*N*-phenacyl-4,5-dimethyyhiazolium bromide); DTNB: 5,5′-dithiobis(2-nitrobenzoic acid); ACh: acetylthiocholine; BCh: butyrylthiocholine; pNPM: *p*-nitrophenyl-α-D-maltohexaoside; pNPG: *p*-nitrophenyl-α-D-glucopyranoside; Gly-Pro-pNA: Gly-Pro-*p*-nitroanilide hydrochloride; KPB: potassium phosphate buffer; FRET: fluorescence resonance energy transfer; ^1^ *Candida rugosa* lipase (type VII, ≥700 unit/mg); ^2^ *Electrophorus electricus* AChE (1000 units/mg); ^3^ equine serum BChE (≥10 units/mg); ^4^ porcine pancreatic α-amylase (type VII, ≥10 unit/mg); ^5^ *Saccharomyces cerevisiae* α-glucosidase (type I, ≥10 U/mg protein); ^6^ human dipeptidyl peptidase-IV (recombinant, ≥10 units/mg).

**Table 4:** Isoflavone profile of ten bean cultivars.

Genera	Cultivars	Isoflavone Profiles (mg/100 g Extract)					
		Daidzein	Daidzin	Glycitein	Glycitin	Genistein	Genistin
*Phaseolus*	38	<LOD	0.18 ± 0.01 ^bC^	ND	ND	0.32 ± 0.01 ^aCD^	ND
	47	ND	<LOD	ND	ND	ND	ND
	59	ND	ND	4.77 ± 0.34 ^D^	ND	ND	ND
	112	<LOD	0.28 ± 0.03 ^bC^	<LOD	ND	0.50 ± 0.02 ^aC^	ND
*Vigna*	107	132.39 ± 12.70 ^aC^	5.25 ± 0.32 ^bC^	ND	0.58 ± 0.02 ^bD^	0.35 ± 0.01 ^bC^	0.77 ± 0.06 ^bC^
	108	185.71 ± 4.41 ^aC^	8.98 ± 0.25 ^bC^	ND	2.59 ± 0.02 ^cD^	0.54 ± 0.01 ^cC^	0.98 ± 0.01 ^cC^
	CN4	12.33 ± 0.65 ^aC^	0.34 ± 0.03 ^bC^	ND	ND	ND	ND
	CN84-1	141.82 ± 2.44 ^bC^	5.46 ± 0.25 ^dC^	208.22 ± 6.87 ^aC^	16.95 ± 0.99 ^cC^	ND	ND
*Glycine*	SJ5	3976.11 ± 244.92 ^bB^	157.85 ± 8.83 ^cB^	6292.22 ± 21.15 ^aA^	411.84 ± 10.21 ^cA^	2.98 ± 0.13 ^cB^	29.06 ± 0.35 ^cB^
	CM60	11156.09 ± 589.64 ^aA^	423.76 ± 22.01 ^cA^	4539.29 ± 310.24 ^bB^	349.16 ± 6.37 ^cB^	31.69 ± 0.56 ^cA^	102.85 ± 6.32 ^cA^

All data were represented as mean ± standard deviation (SD) of triplicate experiments (*n* = 3). Lowercase letters specified significantly different contents of different isoflavone in the same bean cultivar, while different uppercase letters specified significantly different contents of the same isoflavone in different bean cultivars at *p* < 0.05 using one-way analysis of variance (ANOVA) and Duncan’s multiple comparison test. LOD: limit of detection; ND: not detected.

**Table 5:** Phenolic profiles of ten bean cultivars.

Genera	Cultivars	Phenolic Profiles (mg/100 g Extract)					
		Quercetin	Luteolin	Apigenin	Naringenin	Kaempferol	Isorhamnetin
*Phaseolus*	38	<LOD	ND	ND	<LOD	<LOD	0.52 ± 0.04 ^d^
	47	ND	ND	ND	ND	ND	0.92 ± 0.07 ^b^
	59	ND	ND	ND	<LOD	<LOD	ND
	112	9.44 ± 0.57	ND	ND	ND	<LOD	<LOD
*Vigna*	107	<LOD	ND	ND	ND	44.97 ± 0.43	ND
	108	ND	ND	ND	ND	<LOD	0.63 ± 0.04 ^c^
	CN4	ND	ND	ND	ND	<LOD	1.37 ± 0.13 ^a^
	CN84-1	ND	6.22 ± 0.06	<LOD	<LOD	<LOD	ND
*Glycine*	SJ5	ND	ND	ND	0.67 ± 0.06 ^b^	ND	ND
	CM60	ND	ND	ND	3.00 ± 0.11 ^a^	<LOD	ND

All data were represented as mean ± standard deviation (SD) of triplicate experiments (*n* = 3). Superscript letters specified significantly different contents of the same phenolic in different bean cultivars at *p* < 0.05 using one-way analysis of variance (ANOVA) and Duncan’s multiple comparison test. LOD: limit of detection; ND: not detected.

**Table 6:** Inhibitory activities against enzymes relevant to obesity (lipase), type II diabetes (α-amylase, α-glucosidase and dipeptidyl peptidase-IV (DPP-IV)) and Alzheimer’s disease (acetylcholinesterase (AChE), butyrylcholinesterase (BChE) and β-secretase (BACE-1)) of ten bean cultivars compared to enzyme inhibitors (presented as half maximal inhibitory concentration (IC_50_)).

Genera	Cultivars		Inhibitory Activities (%Inhibition)					
		Lipase ^1^	α-Amylase ^2^	α-Glucosidase ^2^	DPP-IV ^2^	AChE ^1^	BChE ^1^	BACE-1 ^3^
*Phaseolus*	38	17.09 ± 1.46 ^b^	31.06 ± 1.24 ^a^	21.39 ± 1.51 ^f^	38.32 ± 2.55 ^d^	ND	14.54 ± 1.37 ^d^	58.23 ± 2.69 ^a^
	47	12.06 ± 1.01 ^c^	25.79 ± 1.47 ^b^	12.32 ± 0.83 ^g^	35.63 ± 3.39 ^d^	ND	12.00 ± 1.13 ^ef^	53.15 ± 1.58 ^bc^
	59	15.99 ± 1.45 ^b^	25.04 ± 1.69 ^b^	47.08 ± 4.67 ^c^	47.33 ± 1.61 ^b^	ND	13.29 ± 1.27 ^de^	56.17 ± 2.93 ^ab^
	112	20.69 ± 0.91 ^a^	14.15 ± 0.38 ^d^	6.43 ± 0.59 ^h^	13.02 ± 1.11 ^e^	ND	15.94 ± 1.13 ^c^	50.42 ± 4.28 ^c^
*Vigna*	107	6.89 ± 0.48 ^e^	30.08 ± 1.33 ^a^	51.17 ± 4.96 ^bc^	37.02 ± 3.37 ^d^	ND	11.58 ± 0.94 ^f^	24.04 ± 2.12 ^e^
	108	21.91 ± 1.63 ^a^	12.53 ± 0.80 ^d^	26.91 ± 2.17 ^e^	11.95 ± 0.83 ^e^	ND	10.69 ± 0.92 ^fg^	16.38 ± 0.74 ^f^
	CN4	ND	12.91 ± 0.87 ^d^	49.32 ± 3.18 ^c^	51.62 ± 3.37 ^a^	ND	10.64 ± 1.04 ^gh^	57.55 ± 2.29 ^a^
	CN84-1	ND	17.26 ± 1.32 ^c^	37.98 ± 3.59 ^d^	47.97 ± 4.27 ^b^	ND	8.25 ± 0.68 ^h^	54.20 ± 0.65 ^abc^
*Glycine*	SJ5	9.57 ± 0.57 ^d^	24.77 ± 2.41 ^b^	59.83 ± 3.96 ^a^	42.44 ± 4.12 ^c^	11.76 ± 1.15 ^b^	23.12 ± 1.47 ^a^	29.83 ± 0.57 ^d^
	CM60	9.57 ± 0.90 ^d^	26.33 ± 2.03 ^b^	54.43 ± 2.32 ^b^	45.62 ± 2.35 ^b^	18.76 ± 1.68 ^a^	19.94 ± 1.93 ^b^	22.95 ± 1.04 ^e^
Orlistat		7.94 µM	NA	NA	NA	NA	NA	NA
Acarbose		NA	14.58 µM	0.53 µM [21]	NA	NA	NA	NA
Saxagliptin		NA	NA	NA	0.27 µM	NA	NA	NA
Donepezil		NA	NA	NA	NA	3.12 µM [22]	2.14 µM [22]	1.31 µM [22]

All data were represented as mean ± standard deviation (SD) of triplicate experiments (*n* = 3). Superscript letters specified significantly different in vitro inhibitory activities of different bean cultivars in the same enzyme assay (*p* < 0.05) using a one-way analysis of variance (ANOVA) and Duncan’s multiple comparison test. ^1^ Final extract concentration = 10 mg/mL; ^2^ Final extract concentration = 12.5 mg/mL; ^3^ Final extract concentration = 2 mg/mL; ND: not detected; NA: not available.

## Data Availability

Data are contained within this article and Supplementary Material.

## Supplementary Materials

The following supporting information can be downloaded at: https://www.mdpi.com/article/10.3390/foods11233905/s1, Supplementary Table S1. Physical appearances of ten bean cultivars; Supplementary Table S2. Fragment ions of six commercial standards of isoflavones using liquid chromatography-electrospray ionization tandem mass spectrometry (LC-ESI-MS/MS) in selective reaction monitoring (SRM) mode; Supplementary Table S3. The validation parameters of six commercial standards of isoflavones using liquid chromatography-electrospray ionization tandem mass spectrometry (LC-ESI-MS/MS) in selective reaction monitoring (SRM) mode; Supplementary Table S4. Isoflavone profile of ten bean cultivars (mg/100 g bean); Supplementary Figure S1. The liquid chromatography-electrospray ionization tandem mass spectrometry (LC-ESI-MS/MS) chromatograms of six commercial standards of isoflavones; Supplementary Figure S2. The liquid chromatography-electrospray ionization tandem mass spectrometry (LC-ESI-MS/MS) chromatograms of twenty-four commercial standards of phenolics; Supplementary Figure S3. The liquid chromatography-electrospray ionization tandem mass spectrometry (LC-ESI-MS/MS) chromatograms of ten bean cultivars including (a) *Phaseolus lunatus* L. cultivar ‘38’, (b) *Phaseolus lunatus* L. cultivar ‘47’, (c) *Phaseolus lunatus* L. cultivar ‘59’, (d) *Phaseolus vulgaris* L. cultivar ‘112’, (e) *Vigna umbellata* (Thunb.) Ohwi and H. Ohashi cultivar ‘107’, (f) *Vigna angularis* (Wild.) Ohwi and Ohashi cultivar ‘108’, (g) *Vigna mungo* (L.) Hepper cultivar ‘CN4’, (h) *Vigna radiata* (L.) Wilczek cultivar ‘CN84-1’, (i) *Glycine max* (L.) Merrill cultivar ‘SJ5’ and (j) *Glycine max* (L.) Merrill cultivar ‘CM60’ using six isoflavone standards as references; Supplementary Figure S4. The liquid chromatography-electrospray ionization tandem mass spectrometry (LC-ESI-MS/MS) chromatograms of ten bean cultivars including (a) *Phaseolus lunatus* L. cultivar ‘38’, (b) *Phaseolus lunatus* L. cultivar ‘47’, (c) *Phaseolus lunatus* L. cultivar ‘59’, (d) *Phaseolus vulgaris* L. cultivar ‘112’, (e) *Vigna umbellata* (Thunb.) Ohwi and H. Ohashi cultivar ‘107’, (f) *Vigna angularis* (Wild.) Ohwi and Ohashi cultivar ‘108’, (g) *Vigna mungo* (L.) Hepper cultivar ‘CN4’, (h) *Vigna radiata* (L.) Wilczek cultivar ‘CN84-1’, (i) *Glycine max* (L.) Merrill cultivar ‘SJ5’ and (j) *Glycine max* (L.) Merrill cultivar ‘CM60’ using twenty-four phenolic standards as references.

## References

[1] Inthachat W., Suttisansanee U., Kruawan K., On-Nom N., Chupeerach C., Temviriyanukul P. (2022). Evaluation of Mutagenicity and Anti-Mutagenicity of Various Bean Milks Using *Drosophila* with High Bioactivation. Foods.

[2] Bean Consumption Per Capita. https://www.helgilibrary.com/indicators/bean-consumption-per-capita/.

[3] Heer M.M., Winham D.M. (2020). Food Behaviors, Health, and Bean Nutrition Awareness among Low-Income Men: A Pilot Study. Int. J. Environ. Res. Public Health.

[4] Sahasakul Y., Aursalung A., Thangsiri S., Wongchang P., Sangkasa-Ad P., Wongpia A., Polpanit A., Inthachat W., Temviriyanukul P., Suttisansanee U. (2022). Nutritional Compositions, Phenolic Contents, and Antioxidant Potentials of Ten Original Lineage Beans in Thailand. Foods.

[5] Abu-Reidah I.M., Arráez-Román D., Lozano-Sánchez J., Segura-Carretero A., Fernández-Gutiérrez A. (2013). Phytochemical Characterisation of Green Beans (*Phaseolus vulgaris* L.) by Using High-performance Liquid Chromatography Coupled with Time-of-flight Mass Spectrometry. Phytochem. Anal..

[6] Wang F., Huang L., Yuan X., Zhang X., Guo L., Xue C., Chen X. (2021). Nutritional, phytochemical and antioxidant properties of 24 mung bean (Vigna radiate L.) genotypes. Food Prod. Process. Nutr..

[7] Zaheer K., Humayoun Akhtar M. (2017). An updated review of dietary isoflavones: Nutrition, processing, bioavailability and impacts on human health. Crit. Rev. Food Sci. Nutr..

[8] Miladinović J., Đorđević V., Balešević-Tubić S., Petrović K., Ćeran M., Cvejić J., Bursać M., Miladinović D. (2019). Increase of isoflavones in the aglycone form in soybeans by targeted crossings of cultivated breeding material. Sci. Rep..

[9] Noncommunicable Diseases. https://www.who.int/news-room/fact-sheets/detail/noncommunicable-diseases.

[10] Tormo M.A., Gil-Exojo I., Romero de Tejada A., Campillo J.E. (2004). Hypoglycaemic and anorexigenic activities of an alpha-amylase inhibitor from white kidney beans (*Phaseolus vulgaris*) in Wistar rats. Br. J. Nutr..

[11] Guo Y., Wu G., Su X., Yang H., Zhang J. (2009). Antiobesity action of a daidzein derivative on male obese mice induced by a high-fat diet. Nutr. Res..

[12] ICH-Q2B Validation of analytical procedures: Methodology. Proceedings of the International Conference on Harmonization of Technical Requirements for the Registration of Drugs for Human Use.

[13] Sirichai P., Kittibunchakul S., Thangsiri S., On-Nom N., Chupeerach C., Temviriyanukul P., Inthachat W., Nuchuchua O., Aursalung A., Sahasakul Y. (2022). Impact of Drying Processes on Phenolics and In Vitro Health-Related Activities of Indigenous Plants in Thailand. Plants.

[14] Pongkunakorn T., Watcharachaisoponsiri T., Chupeerach C., On-nom N., Suttisansanee U. (2017). Inhibitions of key enzymes relevant to obesity and diabetes of Thai local mushroom extracts. Curr. Appl. Sci. Technol..

[15] Promyos N., Temviriyanukul P., Suttisansanee U. (2017). Evaluation of α-glucosidase inhibitory assay using different sub-classes of flavonoids. Curr. Appl. Sci. Technol..

[16] Suttisansanee U., Kunkeaw T., Thatsanasuwan N., Tonglim J., Temviriyanukul P. (2019). The Investigation on Cholinesterases and BACE1 Inhibitory Activities in Various Tea Infusions. Walailak J. Sci. Technol..

[17] Temviriyanukul P., Kittibunchakul S., Trisonthi P., Inthachat W., Siriwan D., Suttisansanee U. (2021). Analysis of Phytonutrients, Anti-Mutagenic and Chemopreventive Effects of Tropical Fruit Extracts. Foods.

[18] Kim I.S. (2021). Current Perspectives on the Beneficial Effects of Soybean Isoflavones and Their Metabolites for Humans. Antioxidants.

[19] Křížová L., Dadáková K., Kašparovská J., Kašparovský T. (2019). Isoflavones. Molecules.

[20] Liu T.T., Liu X.T., Chen Q.X., Shi Y. (2020). Lipase Inhibitors for Obesity: A Review. Biomed. Pharmacother..

[21] Promyos N., Temviriyanukul P., Suttisansanee U. (2020). Investigation of Anthocyanidins and Anthocyanins for Targeting α-Glucosidase in Diabetes Mellitus. Prev. Nutr. Food Sci..

[22] Temviriyanukul P., Kittibunchakul S., Trisonthi P., Kunkeaw T., Inthachat W., Siriwan D., Suttisansanee U. (2022). Mangifera indica ‘Namdokmai’ Prevents Neuronal Cells from Amyloid Peptide Toxicity and Inhibits BACE-1 Activities in a *Drosophila* Model of Alzheimer’s Amyloidosis. Pharmaceuticals.

[23] Dirir A.M., Daou M., Yousef A.F., Yousef L.F. (2022). A review of alpha-glucosidase inhibitors from plants as potential candidates for the treatment of type-2 diabetes. Phytochem. Rev..

[24] Papoutsis K., Zhang J., Bowyer M.C., Brunton N., Gibney E.R., Lyng J. (2021). Fruit, vegetables, and mushrooms for the preparation of extracts with α-amylase and α-glucosidase inhibition properties: A review. Food Chem..

[25] Neumiller J.J., Wood L., Campbell R.K. (2010). Dipeptidyl peptidase-4 inhibitors for the treatment of type 2 diabetes mellitus. Pharmacotherapy.

[26] Sharma K. (2019). Cholinesterase inhibitors as Alzheimer’s therapeutics (Review). Mol. Med. Rep..

[27] Mancini F., De Simone A., Andrisano V. (2011). Beta-secretase as a target for Alzheimer’s disease drug discovery: An overview of in vitro methods for characterization of inhibitors. Anal. Bioanal. Chem..

[28] Zhang J., Ge Y., Han F., Li B., Yan S., Sun J., Wang L. (2014). Isoflavone Content of Soybean Cultivars from Maturity Group 0 to VI Grown in Northern and Southern China. J. Am. Oil. Chem. Soc..

[29] He F.-J., Chen J.-Q. (2013). Consumption of soybean, soy foods, soy isoflavones and breast cancer incidence: Differences between Chinese women and women in Western countries and possible mechanisms. Food Sci. Hum. Wellnesss.

[30] Murphy P.A., Song T., Buseman G., Barua K., Beecher G.R., Trainer D., Holden J. (1999). Isoflavones in retail and institutional soy foods. J. Agric. Food Chem..

[31] Tadera K., Minami Y., Takamatsu K., Matsuoka T. (2006). Inhibition of alpha-glucosidase and alpha-amylase by flavonoids. J. Nutr. Sci. Vitaminol..

[32] Yin Z., Zhang W., Feng F., Zhang Y., Kang W. (2014). α-Glucosidase inhibitors isolated from medicinal plants. Food Sci. Hum. Wellnesss.

[33] Park M.H., Ju J.W., Park M.J., Han J.S. (2013). Daidzein inhibits carbohydrate digestive enzymes in vitro and alleviates postprandial hyperglycemia in diabetic mice. Eur. J. Pharmacol..

[34] Ko K.P., Kim C.S., Ahn Y., Park S.J., Kim Y.J., Park J.K., Lim Y.K., Yoo K.Y., Kim S.S. (2015). Plasma isoflavone concentration is associated with decreased risk of type 2 diabetes in Korean women but not men: Results from the Korean Genome and Epidemiology Study. Diabetologia.

[35] Nanri A., Mizoue T., Takahashi Y., Kirii K., Inoue M., Noda M., Tsugane S. (2010). Soy product and isoflavone intakes are associated with a lower risk of type 2 diabetes in overweight Japanese women. J. Nutr..

[36] Nguyen C.T., Pham N.M., Do V.V., Binns C.W., Hoang V.M., Dang D.A., Lee A.H. (2017). Soyfood and isoflavone intake and risk of type 2 diabetes in Vietnamese adults. Eur. J. Clin. Nutr..

[37] Kim D.H., Jung H.A., Park S.J., Kim J.M., Lee S., Choi J.S., Cheong J.H., Ko K.H., Ryu J.H. (2010). The effects of daidzin and its aglycon, daidzein, on the scopolamine-induced memory impairment in male mice. Arch. Pharm. Res..

[38] Ko Y.H., Kim S.Y., Lee S.Y., Jang C.G. (2018). 6,7,4′-Trihydroxyisoflavone, a major metabolite of daidzein, improves learning and memory via the cholinergic system and the p-CREB/BDNF signaling pathway in mice. Eur. J. Pharmacol..

[39] Lee Y.B., Lee H.J., Won M.H., Hwang I.K., Kang T.C., Lee J.Y., Nam S.Y., Kim K.S., Kim E., Cheon S.H. (2004). Soy isoflavones improve spatial delayed matching-to-place performance and reduce cholinergic neuron loss in elderly male rats. J. Nutr..

[40] Liu R., Xu B. (2015). Inhibitory Effects of Phenolics and Saponins From Commonly Consumed Food Legumes in China Against Digestive Enzymes Pancreatic Lipase and α-Glycosidase. Int. J. Food Prop..

[41] Ngoh Y.-Y., Choi S.B., Gan C.-Y. (2017). The potential roles of Pinto bean (*Phaseolus vulgaris* cv. Pinto) bioactive peptides in regulating physiological functions: Protease activating, lipase inhibiting and bile acid binding activities. J. Funct. Foods.

[42] Frota K.M., Mendonça S., Saldiva P.H., Cruz R.J., Arêas J.A. (2008). Cholesterol-lowering properties of whole cowpea seed and its protein isolate in hamsters. J. Food Sci..

[43] Hamada Y., Ishiura S., Kiso Y. (2012). BACE1 Inhibitor Peptides: Can an Infinitely Small k cat Value Turn the Substrate of an Enzyme into Its Inhibitor?. ACS Med. Chem. Lett..

[44] Huang W.H., Sheng R., Hu Y.Z. (2009). Progress in the development of nonpeptidomimetic BACE 1 inhibitors for Alzheimer’s disease. Curr. Med. Chem..

[45] Lee D.-H., Lee D.-H., Lee J.-S. (2007). Characterization of a new antidementia β-secretase inhibitory peptide from *Saccharomyces cerevisiae*. Enzyme Microb. Technol..

[46] Zhang X., Yu Y., Sun P., Fan Z., Zhang W., Feng C. (2019). Royal jelly peptides: Potential inhibitors of β-secretase in N2a/APP695swe cells. Sci. Rep..

[47] Huang J., Wu D., Wang J., Li F., Lu L., Gao Y., Zhong Z. (2014). Effects of *Panax notoginseng* saponin on α, β, and γ secretase involved in Aβ deposition in SAMP8 mice. Neuroreport.

[48] Karpagam V., Sathishkumar N., Sathiyamoorthy S., Rasappan P., Shila S., Kim Y.J., Yang D.C. (2013). Identification of BACE1 inhibitors from *Panax ginseng* saponins—An Insilco approach. Comput. Biol. Med..

