# Supplementary material for: Phenolic Profiles and Bioactivities of Ten Original Lineage Beans in Thailand
Source: Foods. 2022 Dec 3;11(23):3905. doi: 10.3390/foods11233905 (PMC9738633; doi:10.3390/foods11233905)

foods

MDPI

Supplementary materials

Phenolic Profiles and Bioactivities of Ten Original Lineage Beans in Thailand

Chaowanee Chupeerach [1], Piya Temviriyanukul [1], Sirinapa Thangsiri [1], Woorawee Inthachat [1], Yuraporn Sahasakul [1], Amornrat Aursalung [1], Pitthaya Wongchang [2], Parichart Sangkasa-ad [2], Aphinya Wongpia [2], Auytin Polpanit [3], Onanong Nuchuchua [4] and Uthaiwan Suttisansanee [1,*]

[1] Food and Nutrition Academic and Research Cluster, Institute of Nutrition, Mahidol University, Salaya, Phuttamonthon, Nakhon Pathom 73170, Thailand; chaowanee.chu@mahidol.ac.th (C.C.); piya.tem@mahidol.ac.th (P.T.); sirinapa.tha@mahidol.ac.th (S.T.); woorawee.int@mahidol.ac.th (W.I.); yuraporn.sah@mahidol.ac.th (Y.S.); amornrat.aur@mahidol.ac.th (A.A.)

[2] Biotechnology Research and Development Office, Department of Agriculture Rangsit-Nakorn Nayok, Rangsit (Klong 6), Thanyaburi, Pathum Thani 12100, Thailand; pitthaya@hotmail.com (P.W.); psk50_2003@hotmail.com (P.S.-a.); aphinya.wongpia@gmail.com (A.W.)

[3] Chiang Mai Field Crops Research Center, Department of Agriculture, Nong Han, San Sai District, Chiang Mai 50290, Thailand; auytin1804@hotmail.com

[4] National Nanotechnology Center (NANOTEC), National Science and Technology Development Agency (NSTDA), Klong Luang, Pathum Thani 12120, Thailand; onanong@nanotec.or.th

* Correspondence: uthaiwan.sut@mahidol.ac.th; Tel.: +66-(0)-2800-2380 (ext. 422)

Supplementary Table S1:

Physical appearances of ten bean cultivars.

Scientific names	English common names	Local Thai names	Cultivars	Appearance	Size (mm) *		
					Seed width	Seed length	Seed thickness
Phaseolus lunatus L.	Lima bean	Thua Lima	38	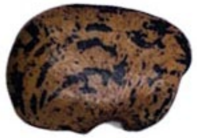	10.04 ± 0.72	14.54 ± 0.87	4.71 ± 1.93
		Thua Kao	47	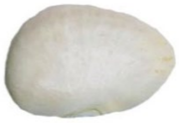	8.56 ± 0.62	12.89 ± 0.66	4.55 ± 1.29
		Thua Boy	59	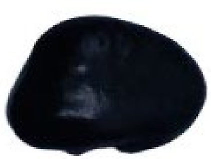	9.87 ± 0.80	14.55 ± 1.39	4.80 ± 1.64
Phaseolus vulgaris L.	Red kidney bean	Thua Daeng Luang	112	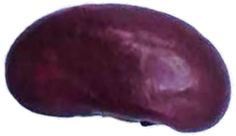	6.25 ± 0.77	16.10 ± 1.18	6.19 ± 0.51
Vigna umbellata (Thunb.) Ohwi & H.Ohashi	Red bean, rice bean	Thua Nio Nang Daeng	107	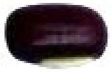	4.60 ± 0.44	7.61 ± 0.53	3.65 ± 0.27
Vigna angularis (Wild.) Ohwi & Ohashi	Azuki bean, adzuki bean	Thua Azuki	108	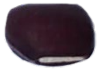	4.93 ± 0.36	7.24 ± 0.58	4.94 ± 0.50

* All data were expressed as mean ± standard deviation (SD) of triplicate experiments (n = 10). Seed sizes were determined using a 0.01mm/0.0005" digital Vernier (Protronics Co., Ltd., Pathum Thani, Thailand)

Supplementary Table S1 (Cont.):

Physical appearances of ten bean cultivars.

Scientific names	English common names	Local Thai names	Cultivars	Appearance	Size (mm) *		
					Seed width	Seed length	Seed thickness
Vigna mungo (L.) Hepper	Black gram, mungo bean	Thua Khiao Phio Dam	CN4	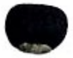	3.84 ± 0.39	4.87 ± 0.47	3.56 ± 0.33
Vigna radiata (L.) Wilczek	Mung bean	Thua Khiao Phio Man	CN84-1	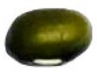	4.03 ± 0.26	5.65 ± 0.62	3.93 ± 0.09
Glycine max (L.) Merrill	Soybean	Thua Lueang	SJ5	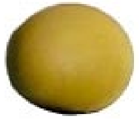	6.00 ± 0.47	7.27 ± 0.47	4.83 ± 0.46
			CM60	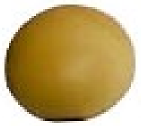	6.53 ± 0.26	7.39 ± 0.32	5.89 ± 0.47

* All data were expressed as mean ± standard deviation (SD) of triplicate experiments (*n* = 10). Seed sizes were determined using a 0.01mm/0.0005" digital Vernier (Protronics Co., Ltd., Pathum Thani, Thailand)

Supplementary Table S2:

Fragment ions of six commercial standards of isoflavones using liquid chromatography–electrospray ionization–tandem mass spectrometry (LC-ESI-MS/MS) in selective reaction monitoring (SRM) mode.

Compounds	Standards	Ion mass	Parent ions (*m*/*z*)	SRM transitions (*m*/*z*) and collision energy (*V*)	RF lens (*V*)
1	Daidzein	[M+H]	255.10	181.125 (30.78 V), 199.125 (25.39 V)	283
2	Daidzin	[M+H]	417.15	199.00 (50 V), 255.125 (18.18 V)	183
3	Glycitein	[M+H]	285.10	242.125 (33.1 V), 270.125 (26.74 V)	299
4	Glycitin	[M+H]	447.15	270.054 (43.54 V), 285.125 (16.08 V)	173
5	Genistein	[M+H]	269.138	132.929 (30.95 V), 159.054 (29.26 V), 224.054 (25.6 V)	239
6	Genistin	[M-H]	433.138	153 (58 V), 271.125 (17.97 V)	179

Supplementary Table S3:

The validation parameters of six commercial standards of isoflavones using liquid chromatography–electrospray ionization–tandem mass spectrometry (LC-ESI-MS/MS) in selective reaction monitoring (SRM) mode.

Compounds	Standards	Retention time (min)	Linear range (µg/mL)	Linear regression equation	Correlation coefficient (R^2)	LOD (µg/mL)	LOQ (µg/mL)	%RSD (Inter-day)	%Recovery		
									Low level (µg/mL)	Medium level (µg/mL)	High level (µg/mL)
1	Daidzein	4.60	0.050–100	y = 31.08x + 78.947	0.9912	4.15	13.82	3.17	109.98	86.34	101.52
2	Daidzin	4.60	0.002–3.125	y = 13198x + 339.75	0.9946	0.04	0.13	3.14	81.16	82.29	106.26
3	Glycitein	4.66	0.005–50	y = 57.457x + 90.296	0.9947	0.14	0.48	8.49	93.53	118.81	101.97
4	Glycitin	4.67	0.003–1.563	y = 6491.9x – 21.535	0.9987	0.09	0.30	2.41	98.88	97.60	105.89
5	Genistein	4.99	0.006–3.125	y = 1786.2x + 11.115	0.9975	0.07	0.23	6.67	88.11	111.67	107.76
6	Genistin	4.66	0.001–0.782	y = 34601x – 119.99	0.9984	0.03	0.10	2.35	86.46	96.08	107.98

LOD: limit of detection; LOQ: limit of quantitation; RSD: relative standard deviation

Supplementary Table S4:

Isoflavone profile of ten bean cultivars (mg/100 g bean).

Cultivars	Isoflavone Profiles (mg/100 g bean)					
	Daidzein	Daidzin	Glycitein	Glycitin	Genistein	Genistin
38	< LOD	0.06 ± 0.01 ^{bC}	ND	ND	0.10 ± 0.00 ^{aC}	ND
47	ND	< LOD	ND	ND	ND	ND
59	ND	ND	1.54 ± 0.24 ^{C}	ND	ND	ND
112	< LOD	0.12 ± 0.01 ^{bC}	< LOD	ND	0.20 ± 0.02 ^{aC}	ND
107	23.83 ± 2.29 ^{aC}	0.95 ± 0.06 ^{bC}	ND	0.11 ± 0.00 ^{bD}	0.06 ± 0.00 ^{bC}	0.14 ± 0.01 ^{bC}
108	53.85 ± 1.28 ^{aC}	2.60 ± 0.07 ^{bC}	ND	0.75 ± 0.01 ^{cD}	0.16 ± 0.00 ^{cC}	0.28 ± 0.00 ^{cC}
CN4	3.24 ± 0.43 ^{aC}	0.09 ± 0.01 ^{bC}	ND	ND	ND	ND
CN84-1	32.62 ± 0.56 ^{bC}	1.26 ± 0.06 ^{dC}	47.89 ± 1.58 ^{aC}	3.90 ± 0.23 ^{cC}	ND	ND
SJ5	1153.07 ± 71.03 ^{bB}	44.17 ± 4.69 ^{dB}	1824.74 ± 6.63 ^{aB}	119.43 ± 2.69 ^{cB}	0.86 ± 0.04 ^{dB}	8.43 ± 0.1 ^{dB}
CM60	6470..53 ± 341.99 ^{aA}	245.78 ± 12.77 ^{cA}	2632.79 ± 179.94 ^{bA}	202.51 ± 3.69 ^{cA}	18.38 ± 0.32 ^{cA}	59.65 ± 3.66 ^{cA}

All data were represented as mean ± standard deviation (SD) of triplicate experiments ($n = 3$). Lowercase letters specified significantly different contents of different isoflavone in the same bean cultivar, while different uppercase letters specified significantly different contents of the same isoflavone in different bean cultivars at $p < 0.05$ using one-way analysis of variance (ANOVA) and Duncan's multiple comparison test. LOD: limit of detection; ND: not detected.

Supplementary Figure S1:

The liquid chromatography–electrospray ionization–tandem mass spectrometry (LC-ESI-MS/MS) chromatograms of six commercial standards of isoflavones.

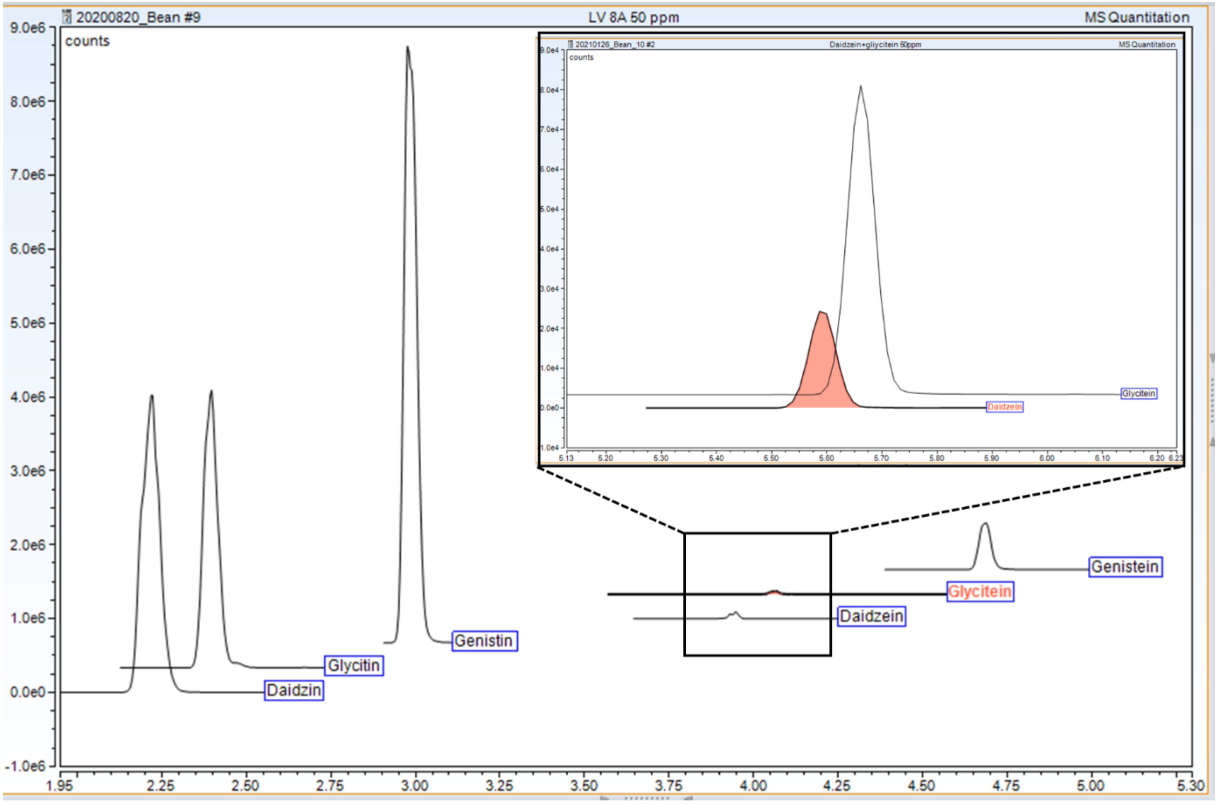

Supplementary Figure S2:

The liquid chromatography–electrospray ionization–tandem mass spectrometry (LC-ESI-MS/MS) chromatograms of twenty-four commercial standards of phenolics.

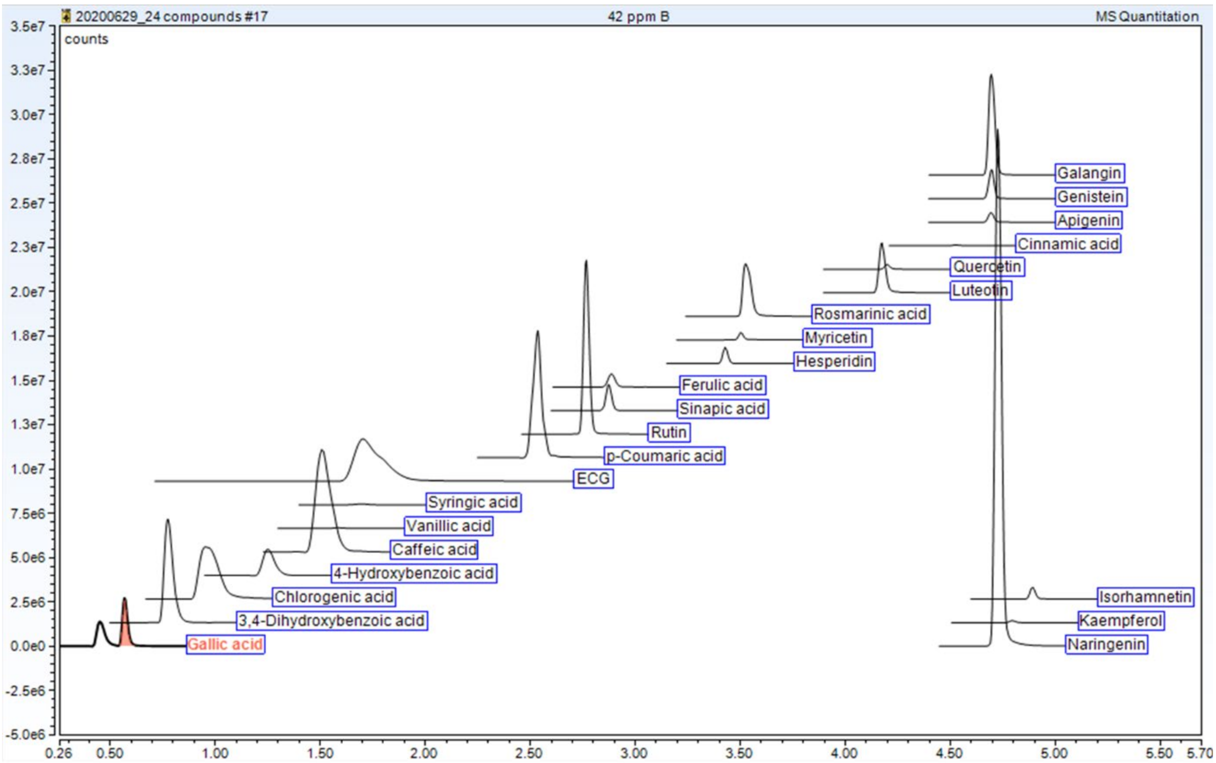

Supplementary Figure S3:

The liquid chromatography–electrospray ionization–tandem mass spectrometry (LC-ESI-MS/MS) chromatograms of ten bean cultivars including (**a**) *Phaseolus lunatus* L. cultivar '38', (**b**) *Phaseolus lunatus* L. cultivar '47', (**c**) *Phaseolus lunatus* L. cultivar '59', (**d**) *Phaseolus vulgaris* L. cultivar '112', (**e**) *Vigna umbellata* (Thunb.) Ohwi & H.Ohashi cultivar '107', (**f**) *Vigna angularis* (Wild.) Ohwi & Ohashi cultivar '108', (**g**) *Vigna mungo* (L.) Hepper cultivar 'CN4', (**h**) *Vigna radiata* (L.) Wilczek cultivar 'CN84-1', (**i**) *Glycine max* (L.) Merrill cultivar 'SJ5' and (**j**) *Glycine max* (L.) Merrill cultivar 'CM60' using six isoflavone standards as references.

(a)

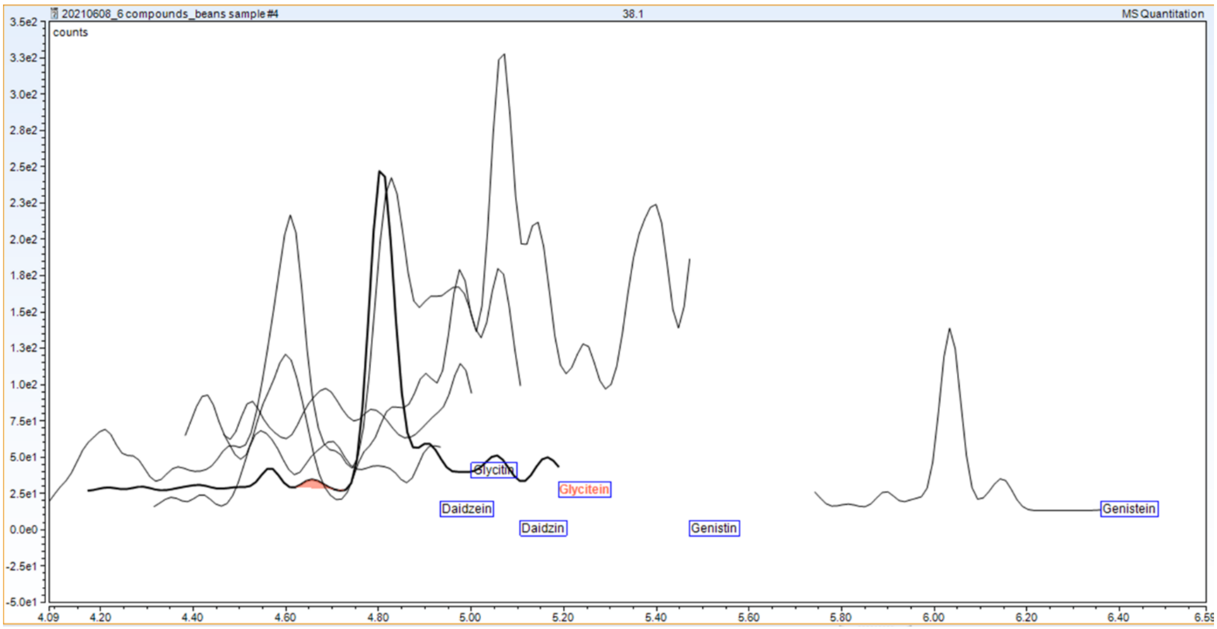

(b)

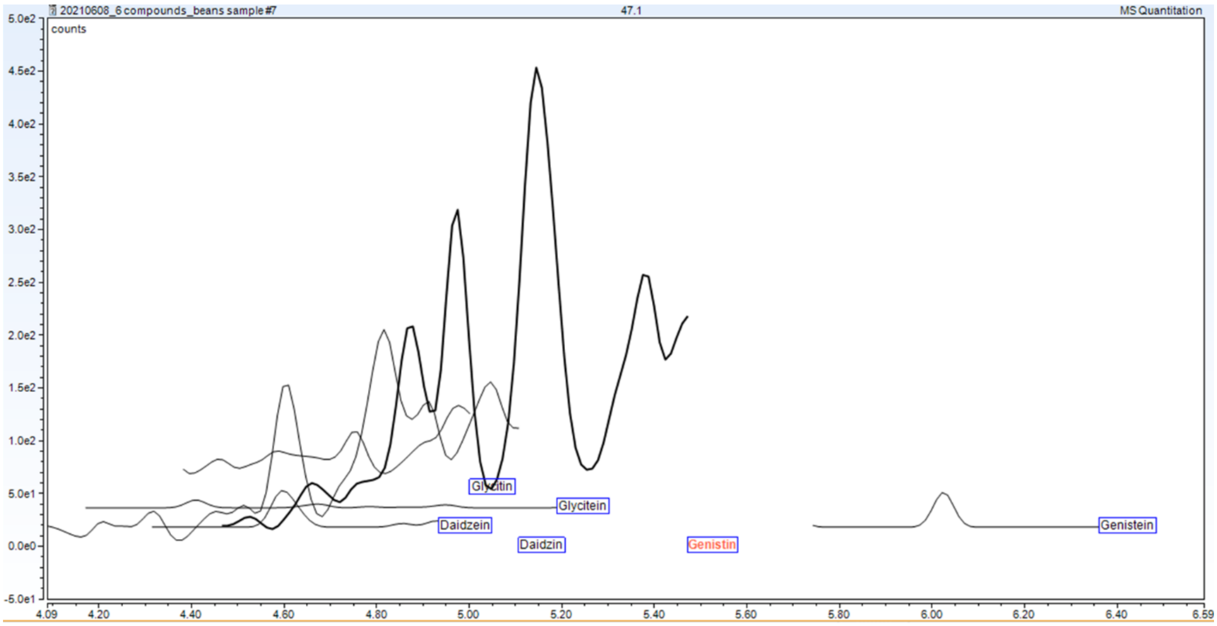

Supplementary Figure S3 (Cont.):

The liquid chromatography–electrospray ionization–tandem mass spectrometry (LC-ESI-MS/MS) chromatograms of ten bean cultivars including (**a**) *Phaseolus lunatus* L. cultivar '38', (**b**) *Phaseolus lunatus* L. cultivar '47', (**c**) *Phaseolus lunatus* L. cultivar '59', (**d**) *Phaseolus vulgaris* L. cultivar '112', (**e**) *Vigna umbellata* (Thunb.) Ohwi & H.Ohashi cultivar '107', (**f**) *Vigna angularis* (Wild.) Ohwi & Ohashi cultivar '108', (**g**) *Vigna mungo* (L.) Hepper cultivar 'CN4', (**h**) *Vigna radiata* (L.) Wilczek cultivar 'CN84-1', (**i**) *Glycine max* (L.) Merrill cultivar 'SJ5' and (**j**) *Glycine max* (L.) Merrill cultivar 'CM60' using six isoflavone standards as references.

(c)

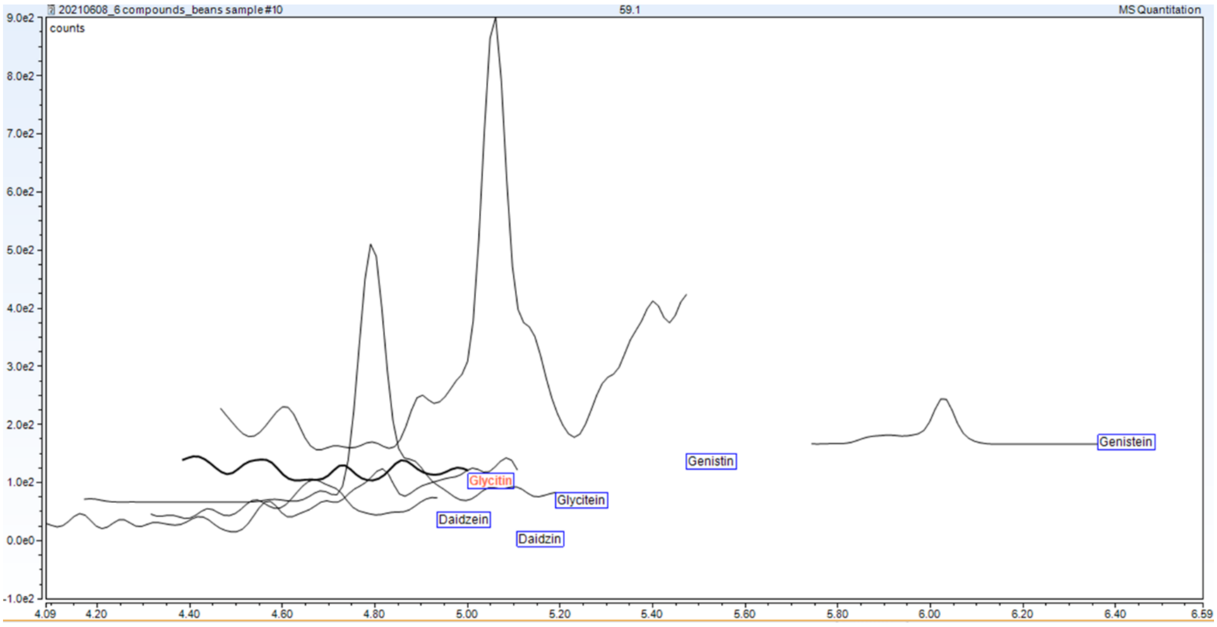

(d)

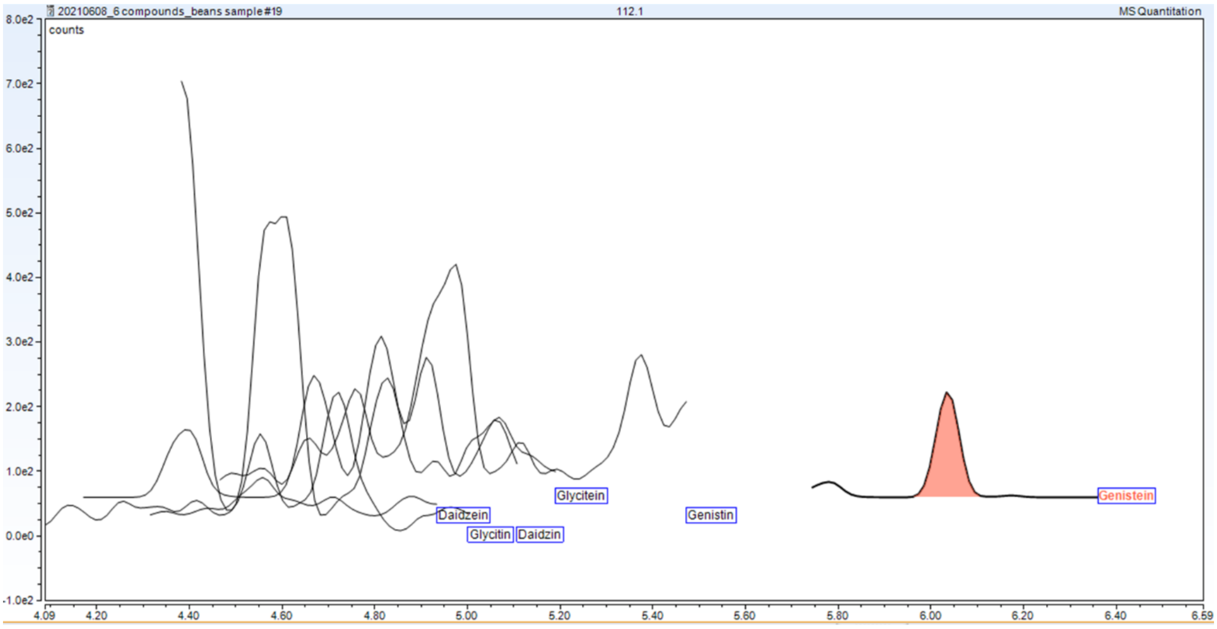

Supplementary Figure S3 (Cont.):

The liquid chromatography–electrospray ionization–tandem mass spectrometry (LC-ESI-MS/MS) chromatograms of ten bean cultivars including (**a**) *Phaseolus lunatus* L. cultivar '38', (**b**) *Phaseolus lunatus* L. cultivar '47', (**c**) *Phaseolus lunatus* L. cultivar '59', (**d**) *Phaseolus vulgaris* L. cultivar '112', (**e**) *Vigna umbellata* (Thunb.) Ohwi & H.Ohashi cultivar '107', (**f**) *Vigna angularis* (Wild.) Ohwi & Ohashi cultivar '108', (**g**) *Vigna mungo* (L.) Hepper cultivar 'CN4', (**h**) *Vigna radiata* (L.) Wilczek cultivar 'CN84-1', (**i**) *Glycine max* (L.) Merrill cultivar 'SJ5' and (**j**) *Glycine max* (L.) Merrill cultivar 'CM60' using six isoflavone standards as references.

(e)

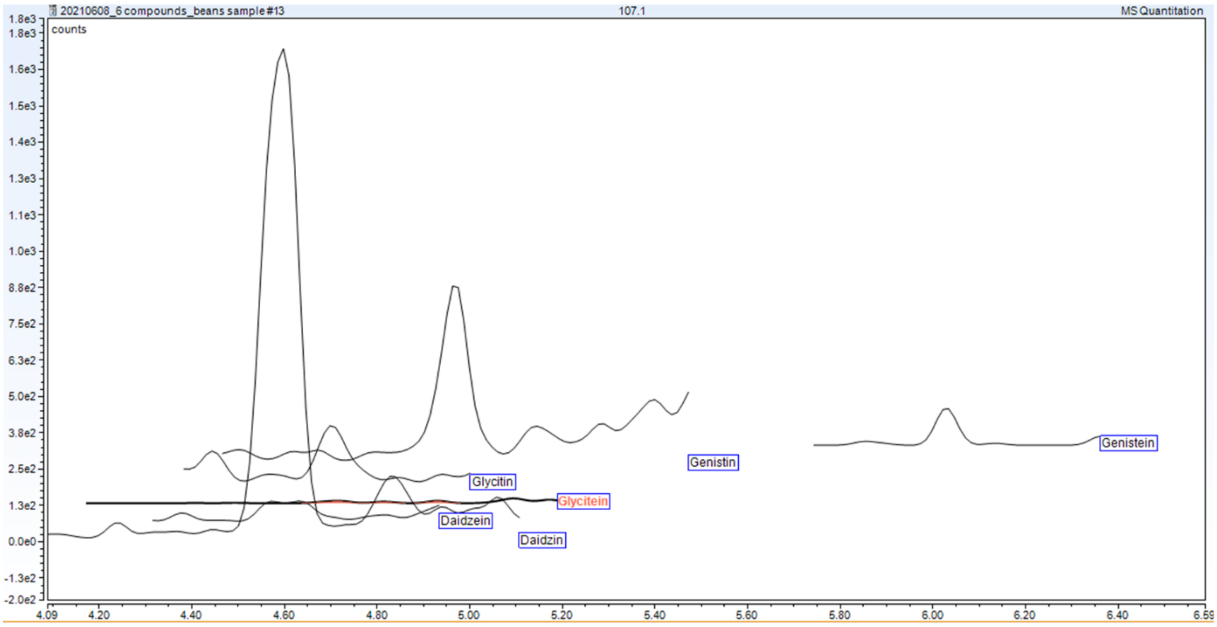

(f)

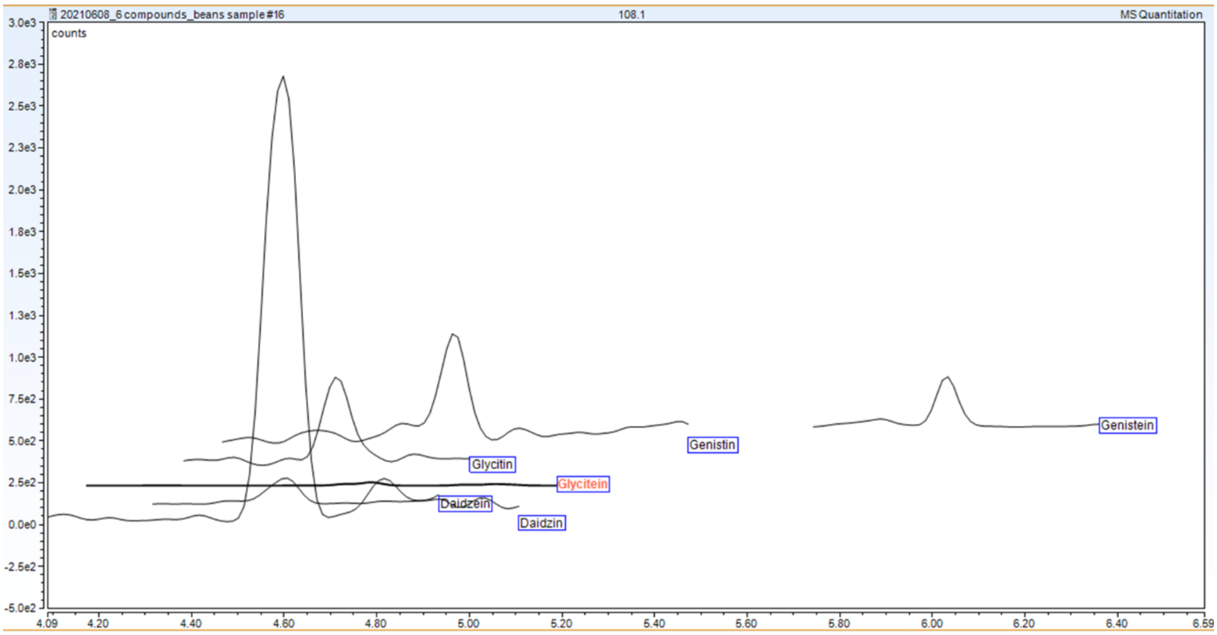

Supplementary Figure S3 (Cont.):

The liquid chromatography–electrospray ionization–tandem mass spectrometry (LC-ESI-MS/MS) chromatograms of ten bean cultivars including (**a**) *Phaseolus lunatus* L. cultivar '38', (**b**) *Phaseolus lunatus* L. cultivar '47', (**c**) *Phaseolus lunatus* L. cultivar '59', (**d**) *Phaseolus vulgaris* L. cultivar '112', (**e**) *Vigna umbellata* (Thunb.) Ohwi & H.Ohashi cultivar '107', (**f**) *Vigna angularis* (Wild.) Ohwi & Ohashi cultivar '108', (**g**) *Vigna mungo* (L.) Hepper cultivar 'CN4', (**h**) *Vigna radiata* (L.) Wilczek cultivar 'CN84-1', (**i**) *Glycine max* (L.) Merrill cultivar 'SJ5' and (**j**) *Glycine max* (L.) Merrill cultivar 'CM60' using six isoflavone standards as references.

(g)

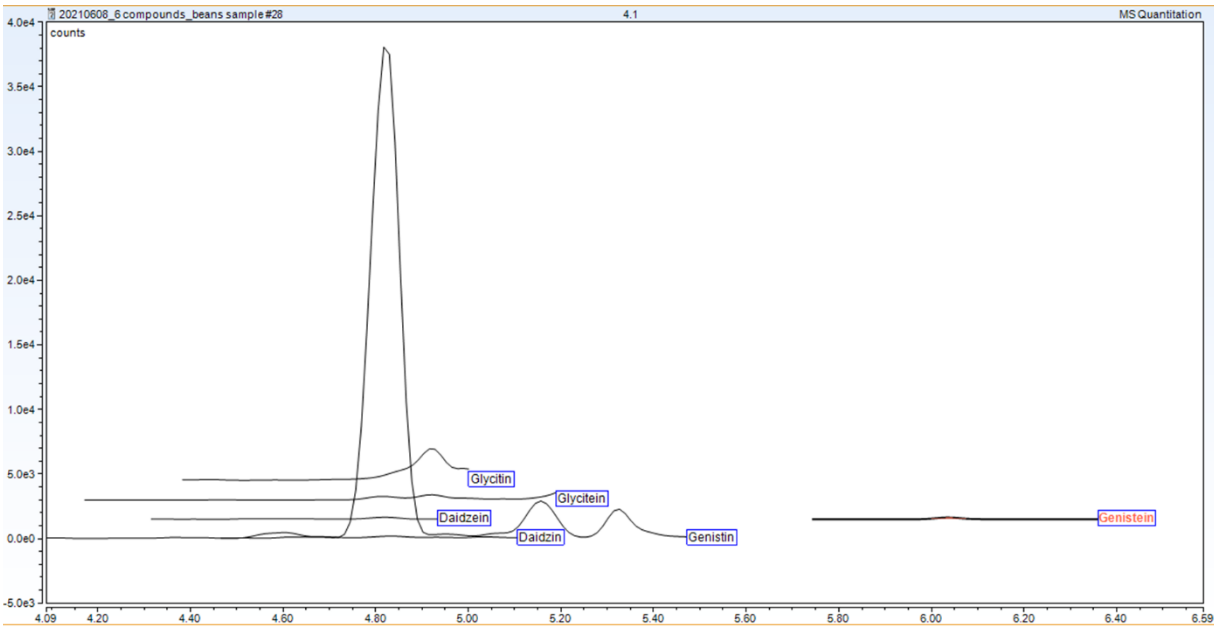

(h)

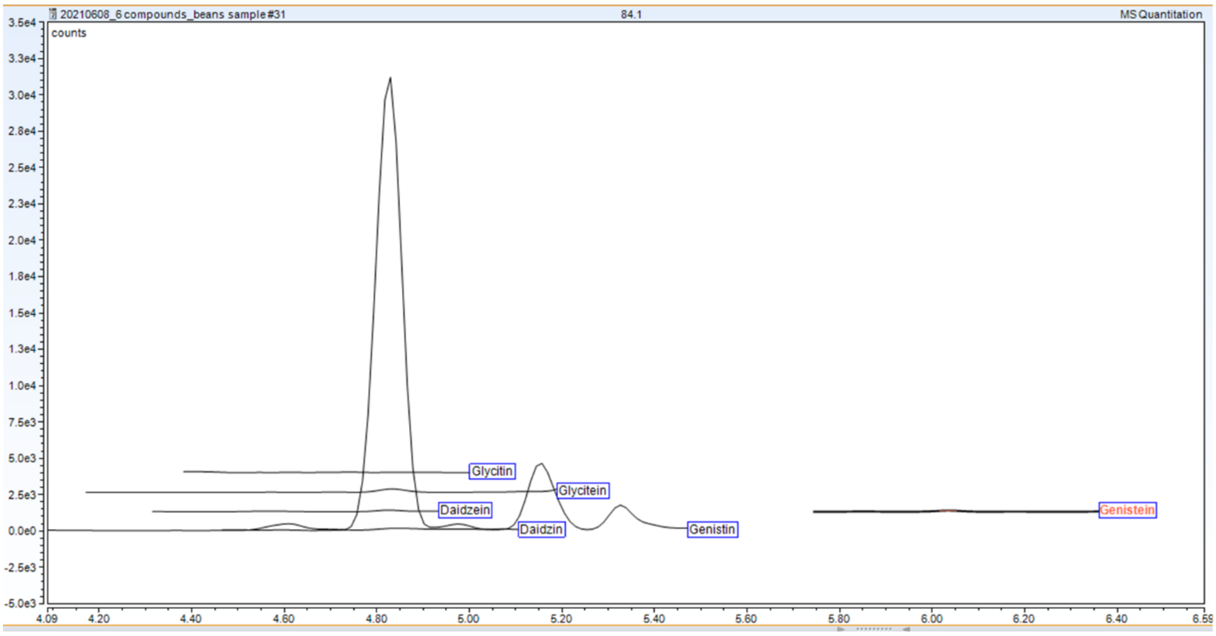

Supplementary Figure S3 (Cont.):

The liquid chromatography–electrospray ionization–tandem mass spectrometry (LC-ESI-MS/MS) chromatograms of ten bean cultivars including (**a**) *Phaseolus lunatus* L. cultivar '38', (**b**) *Phaseolus lunatus* L. cultivar '47', (**c**) *Phaseolus lunatus* L. cultivar '59', (**d**) *Phaseolus vulgaris* L. cultivar '112', (**e**) *Vigna umbellata* (Thunb.) Ohwi & H.Ohashi cultivar '107', (**f**) *Vigna angularis* (Wild.) Ohwi & Ohashi cultivar '108', (**g**) *Vigna mungo* (L.) Hepper cultivar 'CN4', (**h**) *Vigna radiata* (L.) Wilczek cultivar 'CN84-1', (**i**) *Glycine max* (L.) Merrill cultivar 'SJ5' and (**j**) *Glycine max* (L.) Merrill cultivar 'CM60' using six isoflavone standards as references.

(i)

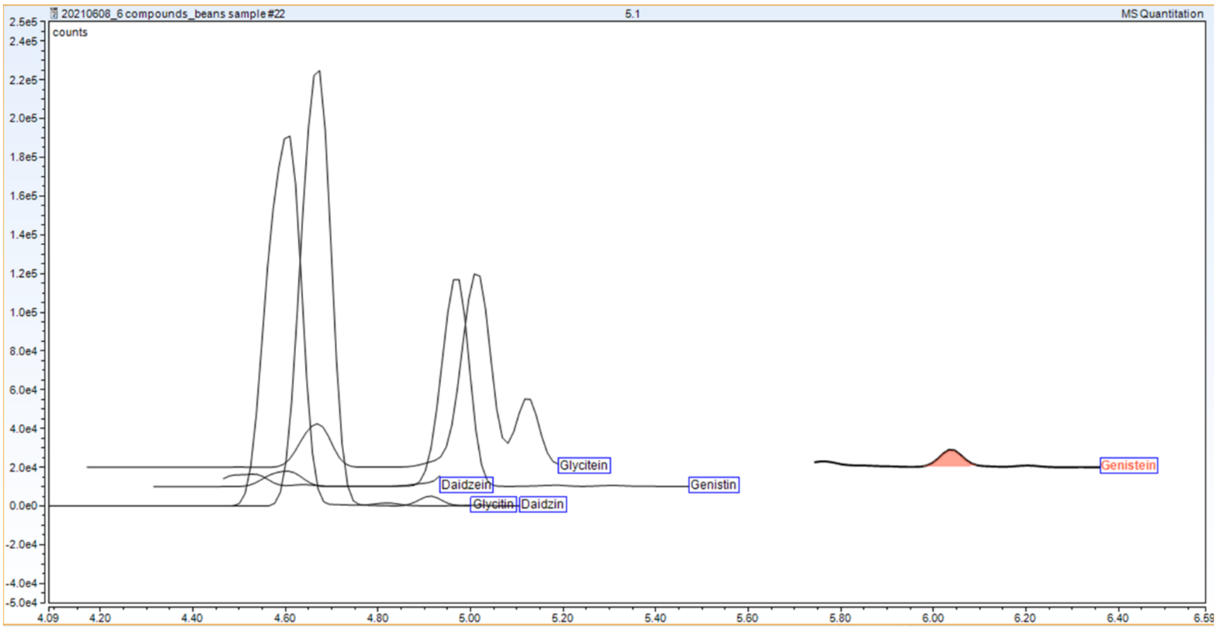

(j)

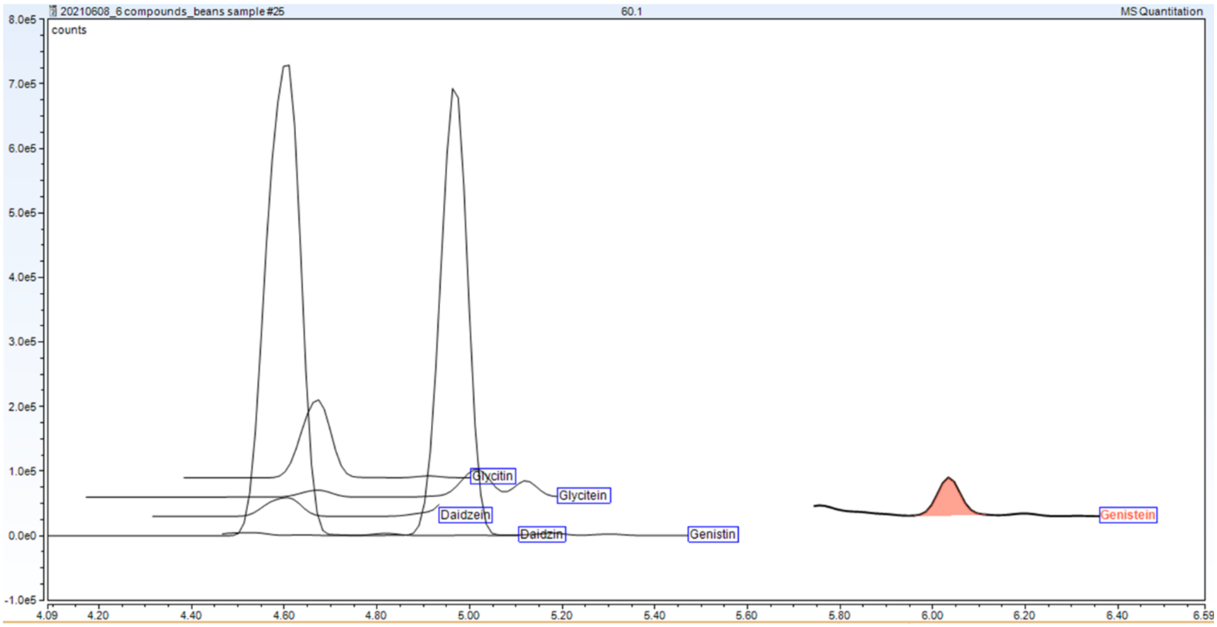

Supplementary Figure S4:

The liquid chromatography–electrospray ionization–tandem mass spectrometry (LC-ESI-MS/MS) chromatograms of ten bean cultivars including (**a**) *Phaseolus lunatus* L. cultivar '38', (**b**) *Phaseolus lunatus* L. cultivar '47', (**c**) *Phaseolus lunatus* L. cultivar '59', (**d**) *Phaseolus vulgaris* L. cultivar '112', (**e**) *Vigna umbellata* (Thunb.) Ohwi & H.Ohashi cultivar '107', (**f**) *Vigna angularis* (Wild.) Ohwi & Ohashi cultivar '108', (**g**) *Vigna mungo* (L.) Hepper cultivar 'CN4', (**h**) *Vigna radiata* (L.) Wilczek cultivar 'CN84-1', (**i**) *Glycine max* (L.) Merrill cultivar 'SJ5' and (**j**) *Glycine max* (L.) Merrill cultivar 'CM60' using twenty-four phenolic standards as references.

(a)

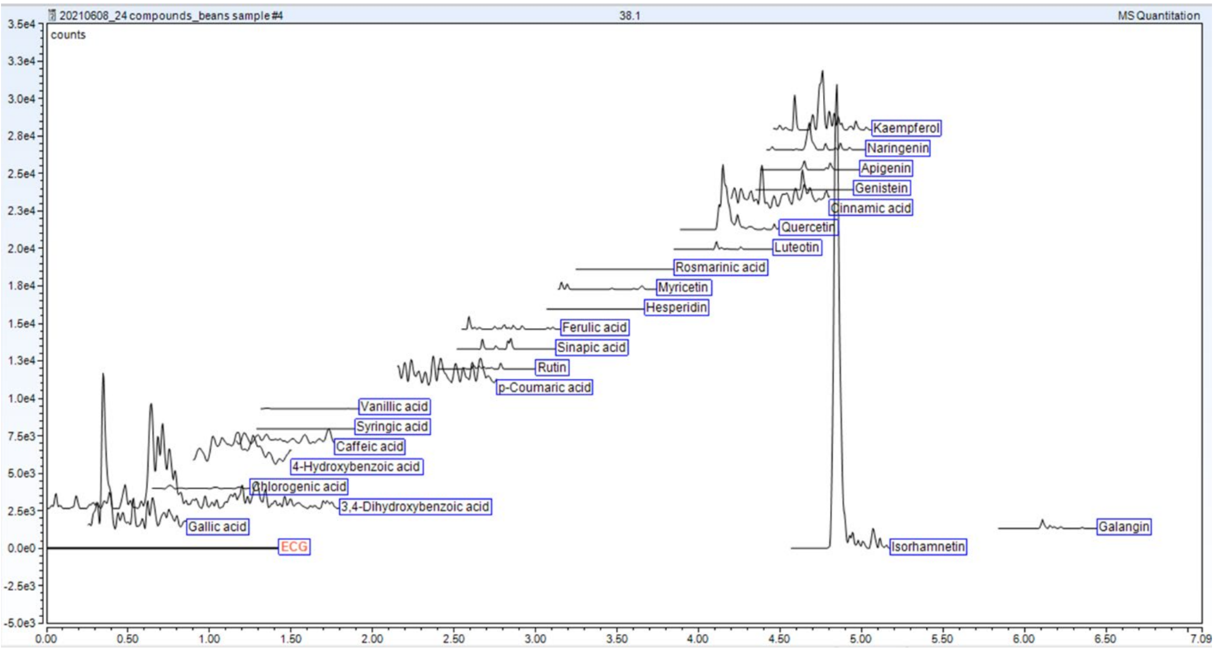

(b)

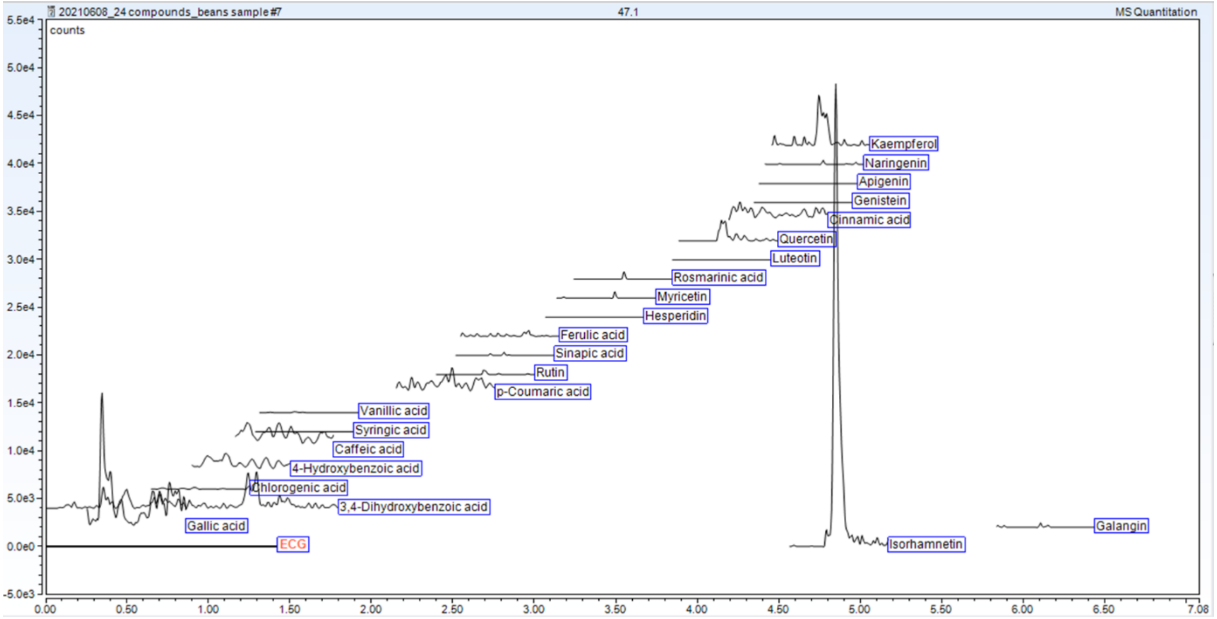

Supplementary Figure S4 (Cont.):

The liquid chromatography–electrospray ionization–tandem mass spectrometry (LC-ESI-MS/MS) chromatograms of ten bean cultivars including (**a**) *Phaseolus lunatus* L. cultivar '38', (**b**) *Phaseolus lunatus* L. cultivar '47', (**c**) *Phaseolus lunatus* L. cultivar '59', (**d**) *Phaseolus vulgaris* L. cultivar '112', (**e**) *Vigna umbellata* (Thunb.) Ohwi & H.Ohashi cultivar '107', (**f**) *Vigna angularis* (Wild.) Ohwi & Ohashi cultivar '108', (**g**) *Vigna mungo* (L.) Hepper cultivar 'CN4', (**h**) *Vigna radiata* (L.) Wilczek cultivar 'CN84-1', (**i**) *Glycine max* (L.) Merrill cultivar 'SJ5' and (**j**) *Glycine max* (L.) Merrill cultivar 'CM60' using twenty-four phenolic standards as references.

(c)

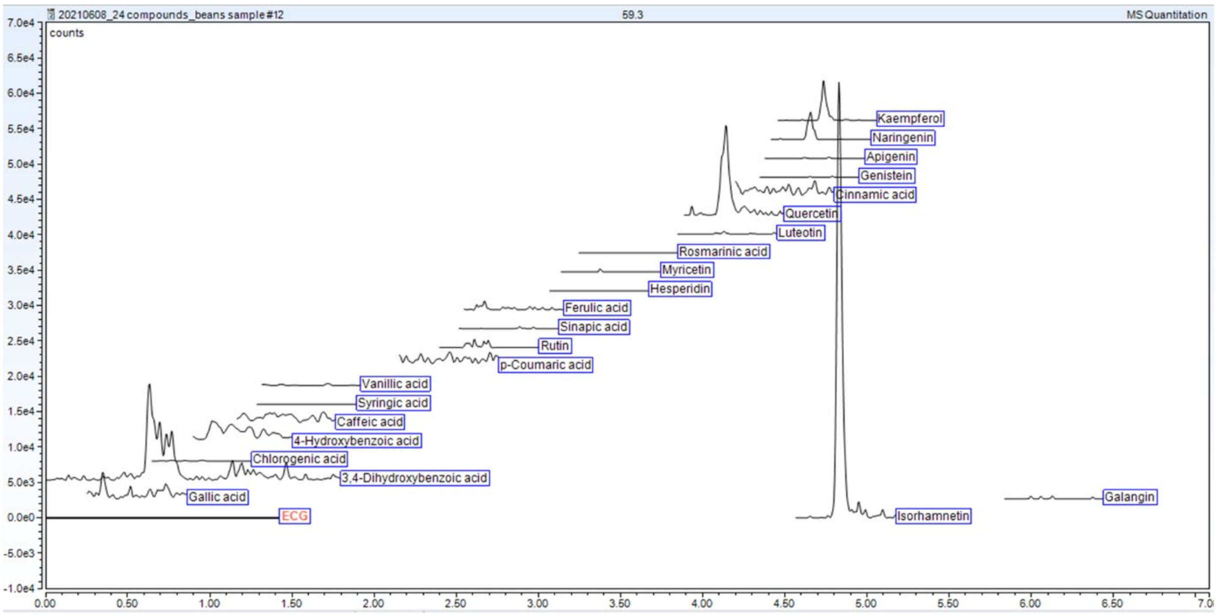

(d)

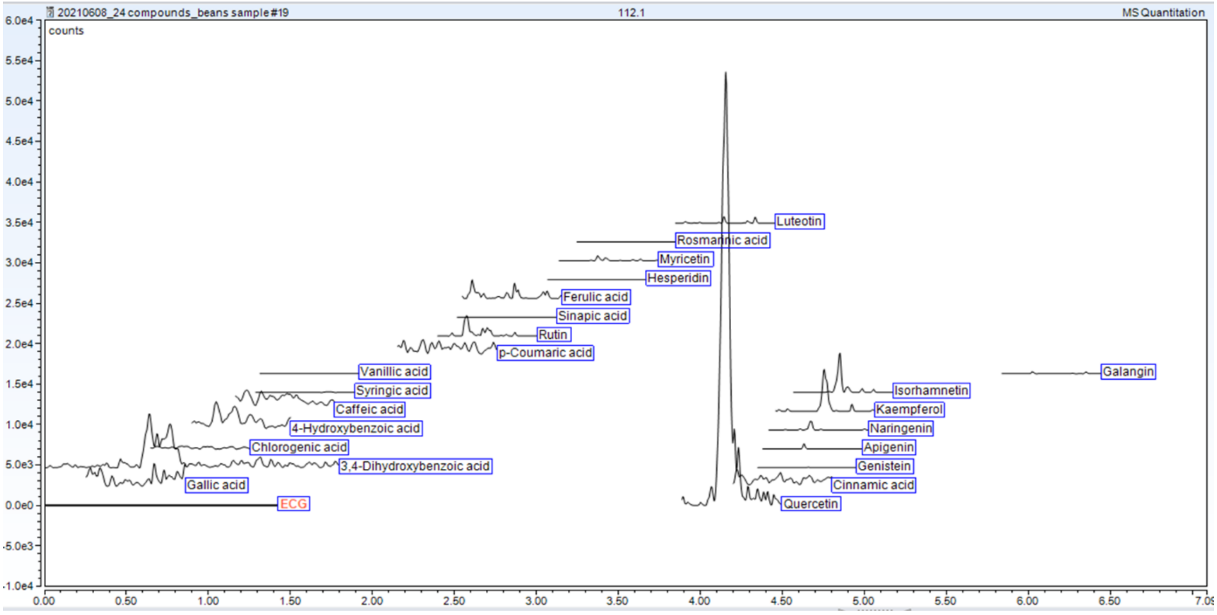

Supplementary Figure S4 (Cont.):

The liquid chromatography–electrospray ionization–tandem mass spectrometry (LC-ESI-MS/MS) chromatograms of ten bean cultivars including (**a**) *Phaseolus lunatus* L. cultivar '38', (**b**) *Phaseolus lunatus* L. cultivar '47', (**c**) *Phaseolus lunatus* L. cultivar '59', (**d**) *Phaseolus vulgaris* L. cultivar '112', (**e**) *Vigna umbellata* (Thunb.) Ohwi & H.Ohashi cultivar '107', (**f**) *Vigna angularis* (Wild.) Ohwi & Ohashi cultivar '108', (**g**) *Vigna mungo* (L.) Hepper cultivar 'CN4', (**h**) *Vigna radiata* (L.) Wilczek cultivar 'CN84-1', (**i**) *Glycine max* (L.) Merrill cultivar 'SJ5' and (**j**) *Glycine max* (L.) Merrill cultivar 'CM60' using twenty-four phenolic standards as references.

(e)

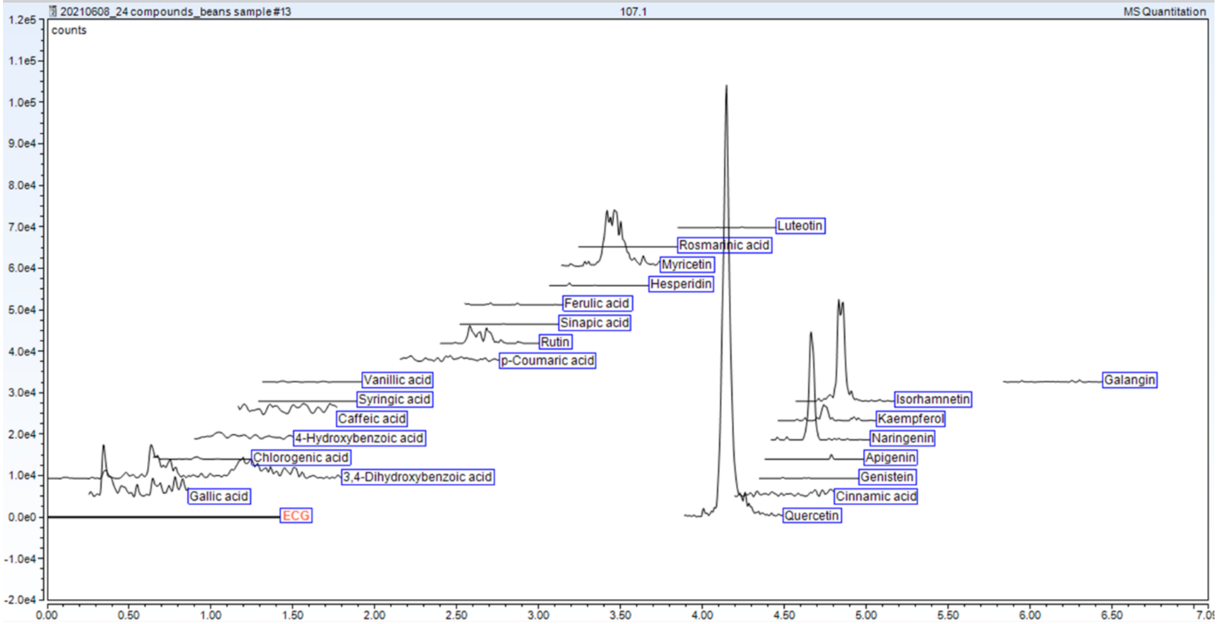

(f)

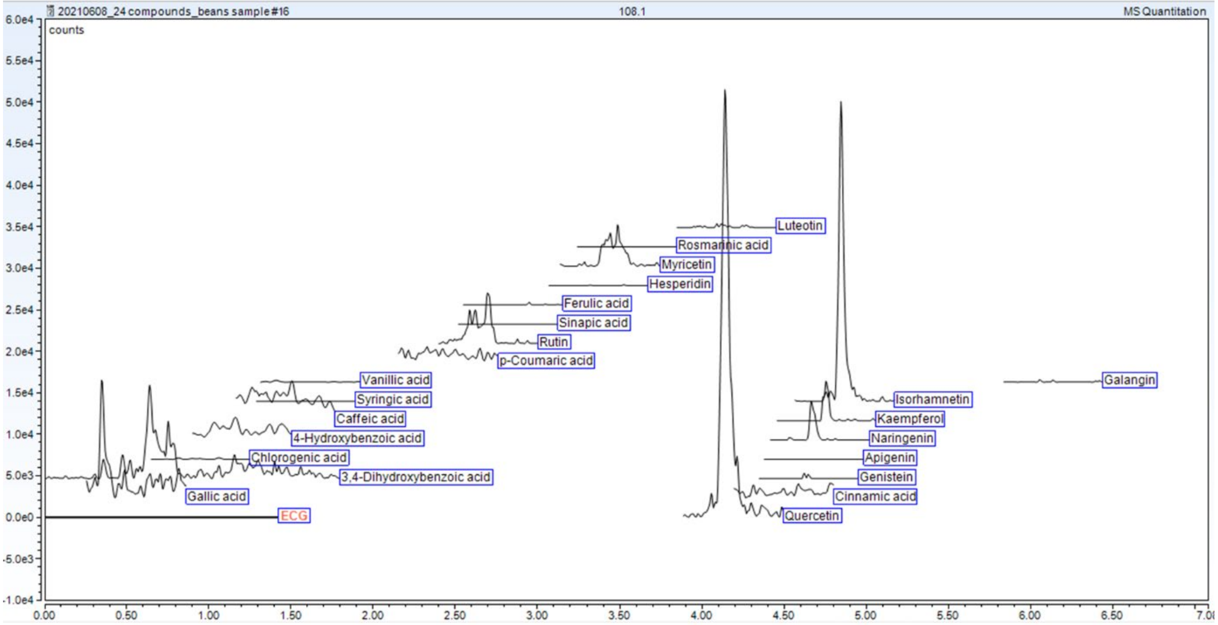

Supplementary Figure S4 (Cont.):

The liquid chromatography–electrospray ionization–tandem mass spectrometry (LC-ESI-MS/MS) chromatograms of ten bean cultivars including (**a**) *Phaseolus lunatus* L. cultivar '38', (**b**) *Phaseolus lunatus* L. cultivar '47', (**c**) *Phaseolus lunatus* L. cultivar '59', (**d**) *Phaseolus vulgaris* L. cultivar '112', (**e**) *Vigna umbellata* (Thunb.) Ohwi & H.Ohashi cultivar '107', (**f**) *Vigna angularis* (Wild.) Ohwi & Ohashi cultivar '108', (**g**) *Vigna mungo* (L.) Hepper cultivar 'CN4', (**h**) *Vigna radiata* (L.) Wilczek cultivar 'CN84-1', (**i**) *Glycine max* (L.) Merrill cultivar 'SJ5' and (**j**) *Glycine max* (L.) Merrill cultivar 'CM60' using twenty-four phenolic standards as references.

(g)

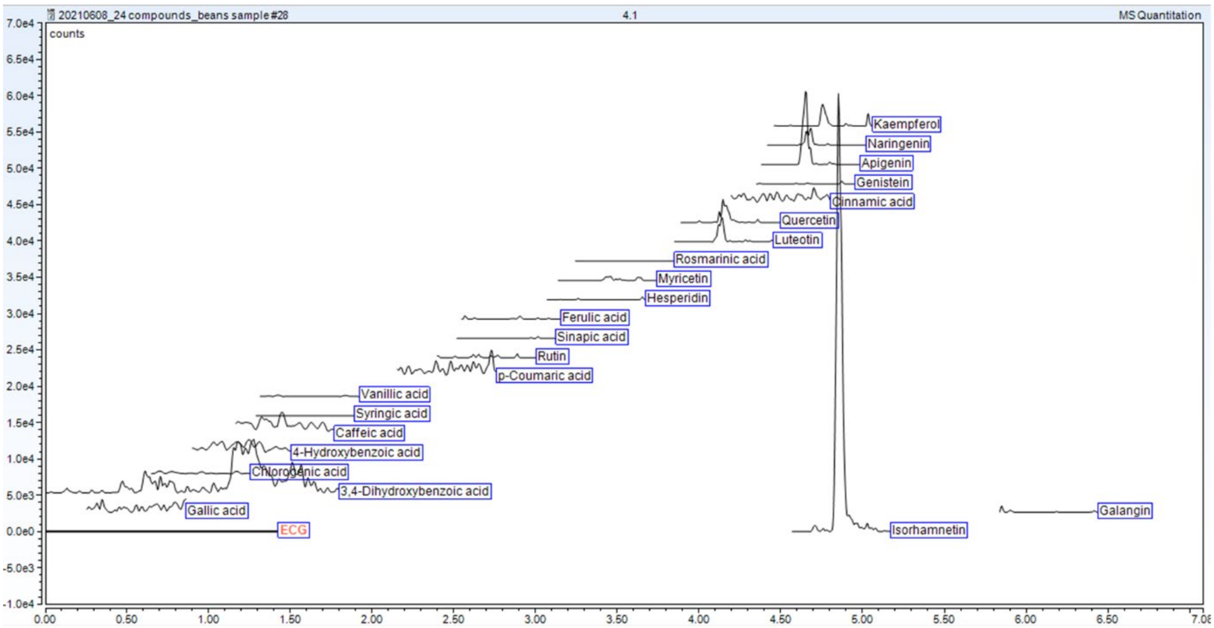

(h)

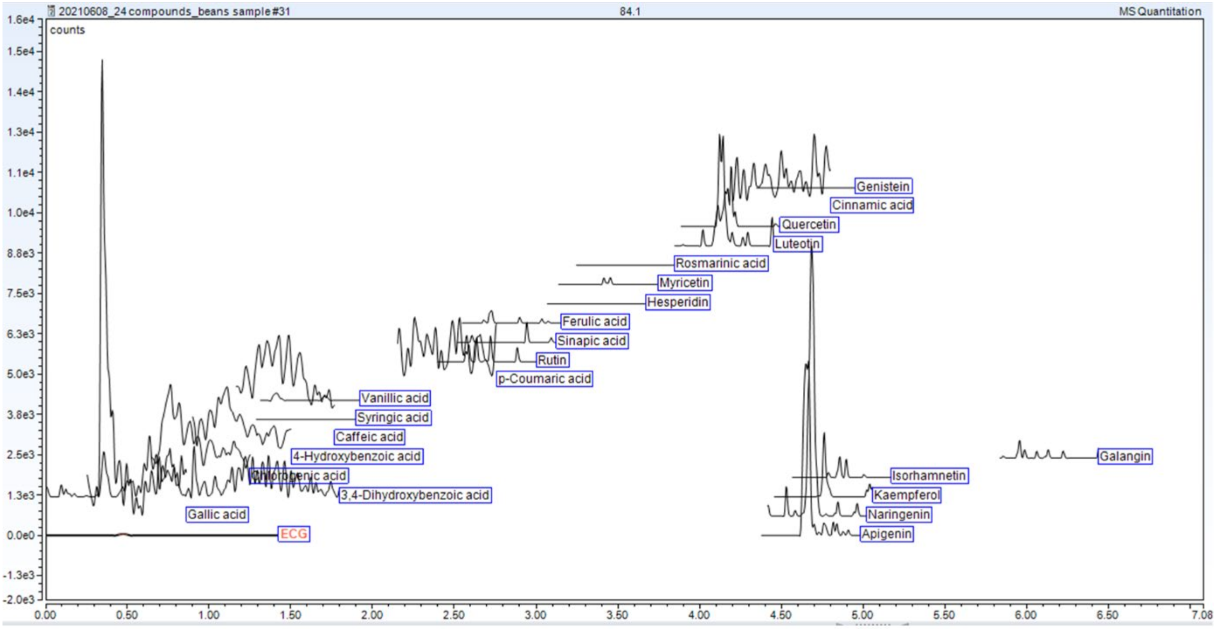

Supplementary Figure S4 (Cont.):

The liquid chromatography–electrospray ionization–tandem mass spectrometry (LC-ESI-MS/MS) chromatograms of ten bean cultivars including (**a**) *Phaseolus lunatus* L. cultivar '38', (**b**) *Phaseolus lunatus* L. cultivar '47', (**c**) *Phaseolus lunatus* L. cultivar '59', (**d**) *Phaseolus vulgaris* L. cultivar '112', (**e**) *Vigna umbellata* (Thunb.) Ohwi & H.Ohashi cultivar '107', (**f**) *Vigna angularis* (Wild.) Ohwi & Ohashi cultivar '108', (**g**) *Vigna mungo* (L.) Hepper cultivar 'CN4', (**h**) *Vigna radiata* (L.) Wilczek cultivar 'CN84-1', (**i**) *Glycine max* (L.) Merrill cultivar 'SJ5' and (**j**) *Glycine max* (L.) Merrill cultivar 'CM60' using twenty-four phenolic standards as references.

(i)

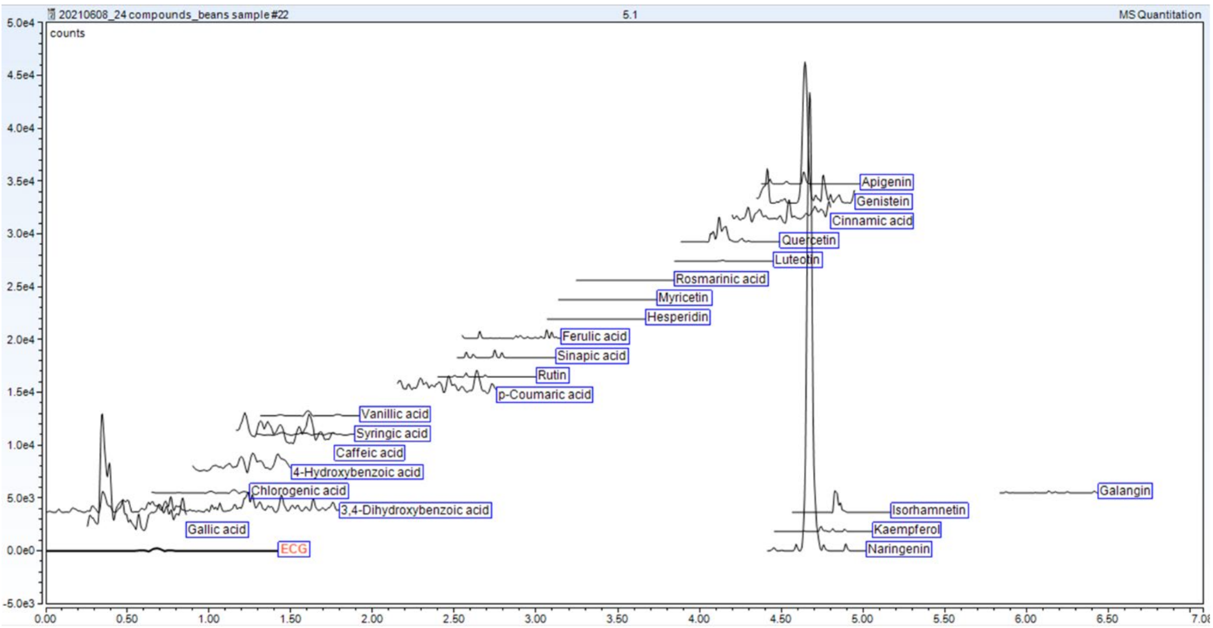

(j)

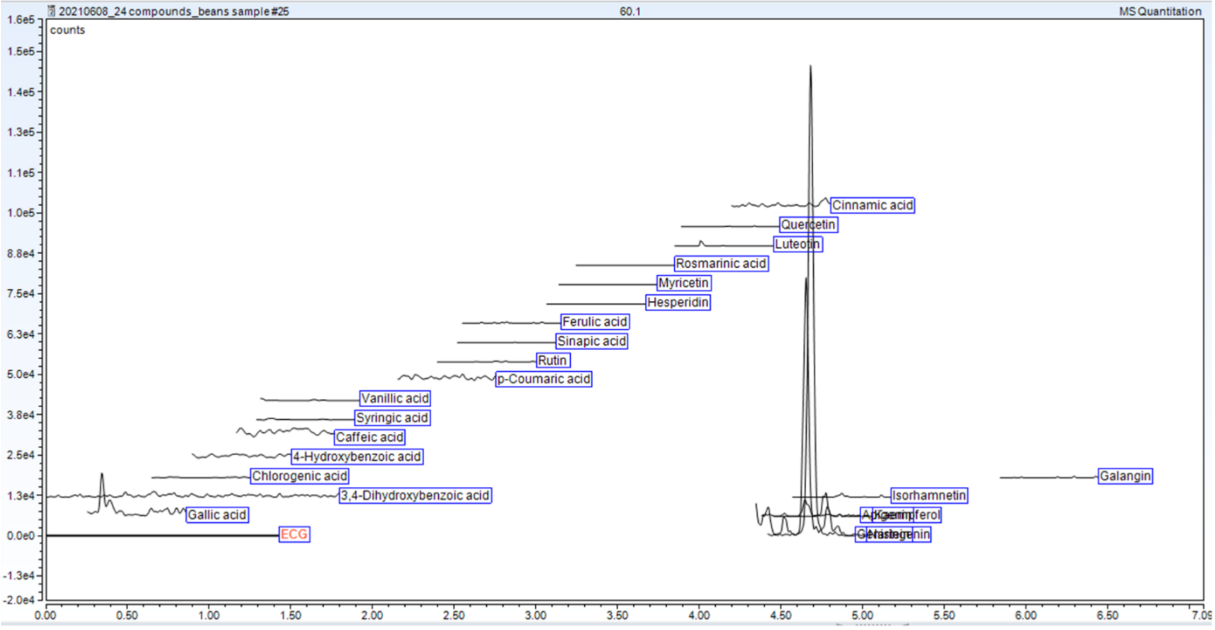

Supplement: Supplementary file 1 [file foods-11-03905-s001.zip › foods-2037613-supplementary.pdf]
